# CD52 Is Elevated on B cells of SLE Patients and Regulates B Cell Function

**DOI:** 10.3389/fimmu.2020.626820

**Published:** 2021-02-04

**Authors:** Kartik Bhamidipati, John L. Silberstein, Yashaar Chaichian, Matthew C. Baker, Tobias V. Lanz, Amin Zia, Yusuf S. Rasheed, Jennifer R. Cochran, William H. Robinson

**Affiliations:** ^1^ Program in Immunology, Stanford University School of Medicine, Stanford, CA, United States; ^2^ VA Palo Alto Healthcare System, Palo Alto, CA, United States; ^3^ Division of Immunology and Rheumatology, School of Medicine, Stanford University, Stanford, CA, United States; ^4^ Department of Bioengineering, Stanford University, Stanford, CA, United States; ^5^ Department of Neurology, Medical Faculty Mannheim, University of Heidelberg, Mannheim, Germany

**Keywords:** systemic lupus erythematosus, B cell receptor signaling, B cells, glycoprotein, autoimmunity

## Abstract

Systemic lupus erythematosus (SLE) is an autoimmune disease characterized by B cell dysregulation and breaks in tolerance that lead to the production of pathogenic autoantibodies. We performed single-cell RNA sequencing of B cells from healthy donors and individuals with SLE which revealed upregulated CD52 expression in SLE patients. We further demonstrate that SLE patients exhibit significantly increased levels of B cell surface CD52 expression and plasma soluble CD52, and levels of soluble CD52 positively correlate with measures of lupus disease activity. Using CD52-deficient JeKo-1 cells, we show that cells lacking surface CD52 expression are hyperresponsive to B cell receptor (BCR) signaling, suggesting an inhibitory role for the surface-bound protein. In healthy donor B cells, antigen-specific BCR-activation initiated CD52 cleavage in a phospholipase C dependent manner, significantly reducing cell surface levels. Experiments with recombinant CD52-Fc showed that soluble CD52 inhibits BCR signaling in a manner partially-dependent on Siglec-10. Moreover, incubation of unstimulated B cells with CD52-Fc resulted in the reduction of surface immunoglobulin and CXCR5. Prolonged incubation of B cells with CD52 resulted in the expansion of IgD+IgM^lo^ anergic B cells. In summary, our findings suggest that CD52 functions as a homeostatic protein on B cells, by inhibiting responses to BCR signaling. Further, our data demonstrate that CD52 is cleaved from the B cell surface upon antigen engagement, and can suppress B cell function in an autocrine and paracrine manner. We propose that increased expression of CD52 by B cells in SLE represents a homeostatic mechanism to suppress B cell hyperactivity.

## Introduction

Systemic lupus erythematosus (SLE) is an autoimmune disease characterized by loss of B cell tolerance that results in the production of pathogenic auto-antibodies and immune complexes, resulting in end organ damage (1, 2). A number of dysregulated pathways have been identified in B cells derived from SLE patients, including increased expression of pro-inflammatory cytokines such as IL-6 and decreased expression of inhibitory receptors such as FCγRIIb [(3), p. 6; (4), p.; (5, 6)]. Previous studies suggested that B cells from SLE patients were more responsive to antigen-specific signaling, driving B cell hyperactivity and autoimmunity (7, 8). However, several recent studies have established that B cells from SLE patients are in fact hyporesponsive to B cell receptor (BCR) signaling and TLR9 signaling, which is hypothesized to reflect a form of B cell anergy acquired due to chronic antigen stimulation in the absence of secondary signals [(9), p. 9; (10)].

In order to identify additional features that differentiated SLE patient-derived B cells from those of healthy donors, we performed single-cell RNA-sequencing on B cells from healthy control (HC) individuals and SLE patients and compared their gene expression profiles. Among several genes found to be differentially expressed across subsets, we identified CD52 as one of the most upregulated genes.

CD52 is a 12 amino acid GPI-linked protein with a large N-linked glycan moiety, expressed widely across immune cells including T and B cells, monocytes, macrophages and eosinophils (11, 12). It is proposed to act as an adhesion molecule and as a complement inhibitory protein [(13), p. 52; (14, 15)]. It is the target of the of the lymphocyte depleting antibody alemtuzumab [(16), p. 52; (17)]. Recent studies demonstrated its role in immune homeostasis in T cells and monocytes [(18), p. 52; (19)]. Upon antigen stimulation, CD52 is cleaved from the T cell surface, which generates a soluble form of CD52 that suppresses T cell receptor signaling through ligation of Siglec-10 [(18, 20), p. 1]. In monocytes, macrophages and dendritic cells, soluble CD52 was found to inhibit NF-κB mediated signaling, with higher concentrations inducing apoptosis (19). The development of secondary autoimmune diseases in a subset of MS patients treated with B and T cell depleting alemtuzumab suggests an important role for CD52 in maintaining immune cell homeostasis (21–23). Nevertheless, to date the role of CD52 in B cell function has not been defined.

Here, we describe our finding that both B cell surface and soluble plasma CD52 are elevated in SLE patients, and that soluble plasma CD52 levels correlate positively with clinical measures of disease activity. Further, we demonstrate that CD52 is cleaved from the surface of B cells upon antigen stimulation and that both the surface and soluble forms inhibit BCR signaling and function, demonstrating a regulatory role for the protein. Our finding suggest that upregulation of surface and soluble CD52 is an autoregulatory mechanism that limits B cell responsiveness to antigen stimulation in SLE.

## Materials and Methods

### Study Subjects

SLE patients (n = 40) were recruited from the Stanford Lupus Clinic. All patients fulfilled at least 4 of the classification criteria for SLE set by the American College of Rheumatology; disease activity was measured using SLE Disease Activity Index (SLEDAI) 2K (24). Relevant clinical information for the patients is listed in **Supplementary Table 1**. Additionally, healthy control (HC) patients (n = 20) were recruited through the Stanford Blood Center to match the demographics of SLE patients. The study was approved by Stanford’s institutional review board and written informed consent was obtained from all participants of the study.

### Isolation of Peripheral Blood Mononuclear Cells, B cells, and Plasma

Whole blood from SLE patients and healthy controls was collected into sodium-heparin tubes (BD Biosciences, San Jose, CA, USA) and diluted 1:1 with PBS prior to isolation of peripheral blood mononuclear cells (PBMCs). PBMCs were purified by density gradient using Ficoll-Paque (GE Healthcare Life Science, Marlborough, MA, USA) in Leucosep tubes (Greiner Bio One, Monroe, LA, USA) per protocols provided by the manufacturer. Blood plasma was collected after centrifugation of blood. The PBMC layer was extracted and washed twice with PBS prior to cryopreservation in Recovery Cell Culture Freezing Medium (Gibco, Grand Island, NY, USA) at 10^7^ PBMCs per ml. PBMC vials were frozen at -80°C overnight prior to long-term storage in liquid nitrogen. Plasma was aliquoted and stored at -80°C until further use. For *in vitro* experiments, B cells were isolated from PBMCs derived from healthy donor whole blood buffy coats available from the Stanford Blood Center. B cells were subsequently isolated using the Human B Cell Isolation Kit (Stemcell Technologies, Vancouver, BC, Canada) according to manufacturer’s protocol. Purity and viability was >97% as assessed by flow cytometry.

### Single Cell RNA-Sequencing

B cells from 2 HC and 3 SLE patients were isolated from PBMCs by EasySep Human Pan-B Cell Enrichment Kit (Stemcell Technologies, Vancouver, BC, Canada) according to manufacturer’s protocols. ~5,000 negatively selected B cells from each of the samples were separately loaded in to the 10X chromium controller (10X Genomics, 95 Pleasanton, CA, USA) for single-cell barcoding. Single cell gene expression libraries were prepared using the Chromium single cell 5’ Reagent Kit per the manufacturer’s protocol and subsequently sequenced on Illumina HiSeq with 100x100 paired end reads (Illumina, San Diego, CA, USA). Base calls were converted to fastq sequences and demultiplexed using the cellranger mkfastq function from 10X Genomics 2.2.0 and aligned to the GRCh38 genome supplied by 10X genomics. Sparse count matrices, barcode assignments and feature calls were prepared using the cellranger count function. Seurat v3.2 was used for gene expression analysis. Cells with less than 50 genes detected, mitochondrial content above 20% of all transcripts or expressing detectable CST3, CD3E, KLRB1, NKG7, and GATA2 transcripts (to remove non-B cell populations like T cells and myeloid cells) were excluded. Next, HC and SLE B cells across patients were merged with other cells from their respective groups into two separate objects, and then subsequently integrated with each other using canonical correlation analysis *via* the FindIntegrationAnchors function (n = 15,039 cells). The integrated data was normalized, scaled, clustered, and then visualized using UMAP. The FindMarkers function was used to identify differentially expressed genes between HC and SLE B cells within each cluster or group of clusters. B cell subsets were annotated using canonical B Cell subset genes such as CD27, FCER2, IGHD, IGHG1, TBX21 and ITGAX. Data is available at accession GSE163121. Publicly available log-normalized gene expression matrices retrieved from Seurat objects were used to correlate expression of CD52 with other detected genes on a single-cell level.

### Flow Cytometry

SLE and HC PBMC samples were thawed and washed in 10 ml of pre-warmed RPMI with 10% FBS. Cells were pelleted by centrifugation at 400 x g for 5 min and then stained with Fixable Viability Stain 510 (BD Biosciences, San Jose, CA, USA) in PBS, blocked with 1 µL Trustain FcX (Biolegend, San Diego, CA, USA) in 50 µL of staining buffer (2% FBS in PBS) and then subsequently stained with two different panels of antibodies in 50 µL in 5 ml polystyrene tubes. One panel included antibodies for CD3, CD14 and CD19 to assess immune cell type expression of CD52 (clone 4C8) and another included B cell phenotyping markers IgD, CD27 and Siglec-10 (clone 5G6) for determination of B cell subset specific expression (25, 26) (Biolegend, San Diego, CA, USA). Once stained, cells were washed, resuspended in PBS with 2% FBS and analyzed using LSR II (BD, San Jose, CA, USA). For *in vitro* experiments, a similar protocol was adapted using 96 well round bottom plates for cell staining. For detection of apoptosis, Pacific Blue™ Annexin V Apoptosis Detection Kit with PI (Biolegend, San Diego, CA, USA) was used according to manufacturer protocols. Antibody clones used for staining are listed in **Supplementary Table 2**.

### Phospholipase C Treatment

Thawed and washed PBMCs (~2.5*10^6^ cells) were resuspended in 1 ml of PBS and incubated at 37°C for 30 min with 1 unit of Phosphatidylinositol-Specific Phospholipase C (PLC)(Thermo Fisher Scientific, Waltham, MA, USA). Cells were subsequently washed and stained with antibodies against CD3, CD14 and CD19 and CD52 (Biolegend, San Diego, CA, USA) and analyzed using a BD LSR II flow cytometer (BD, San Jose, CA, USA).

### Plasma CD52, Immunoglobulin and α2,3 sialylation Quantification

Plasma concentrations of IgG, IgM and soluble CD52 were quantified by enzyme-linked immunosorbent assay (ELISA) according to the manufacturer’s protocols (Human CD52 ELISA kit, Aviva Systems Biology, San Diego, CA, USA; Human IgG ELISA quantitation set, Bethyl Laboratories, Montgomery, TX, USA; Human IgM ELISA quantitation set, Bethyl Laboratories, Montgomery, TX, USA). For quantitation of soluble CD52, plasma samples were diluted 1:10. Duplicates were measured for each sample. The optical density values were obtained on a SpectraMax M3 (Molecular Devices, San Jose, CA, USA) at wavelength of 450 nm. Quantification of α2,3 sialylation was determined as previously described (20). Briefly, plates were coated with MAA-II (Vector Labs, Burlingame, CA, USA) at 20 µg/ml overnight at 4°C, washed twice with PBS, blocked for 1 h at RT with 1% BSA in PBS and plated with 20 µg/ml of CD52-Fc or control Fc prior to incubation with anti-IgG Fc HRP antibody (Bethyl Laboratories, 1:1000 dilution). After two washes with PBS, TMB was added and color development stopped by addition of 0.5M H_2_SO_4_.

### Gene Knockout Pool

CRISPR-Cas9 mediated knockout cell pool of CD52 in JeKo-1 cells were generated by Synthego Corporation (Redwood City, CA, USA). To generate these cells, Ribonucleoproteins containing the Cas9 protein and synthetic chemically modified sgRNA were electroporated into the cells using Synthego’s optimized protocol. Editing efficiency was assessed upon recovery, 48 h post-electroporation. Genomic DNA was extracted from a portion of the cells, PCR amplified and sequenced using Sanger sequencing. The resulting chromatograms were processed using Synthego Inference of CRISPR edits software (ice.synthego.com). Cells were maintained in B cell Media (RPMI supplemented with 10% FBS, 100 U/ml Penicillin, 100 µg/ml Streptomycin, 55 uM B-mercaptoethanol, 2 mM L-glutamine and 5 mM HEPES). Cells were passaged once prior to downstream *in vitro* experiments (P3).

### Cloning, Expression, and Purification of hCD52-Fc and Control Fc

DNA encoding the signal peptide and extracellular portion of human CD52 (amino acids 1–36), fused to a mutant human IgG1 Fc (LALA-PG mutations) through a GGSGG linker and Factor Xa cleavage sequence, was ordered from Integrated DNA Technologies (Coralville, IA) and cloned into a PstI/BamHI digested gWIZ vector (Genlantis, San Diego, CA, USA) using Gibson Assembly (27, 28). At the c-terminus of the Fc, a hexahistidine tag was also added for purification with immobilized metal affinity chromatography. For control Fc, DNA encoding GGSGG linker with Factor Xa cleavage sequence fused to mutant human IgG1 Fc and a hexahistidine tag was cloned into the gWIZ vector with a BM40 signal peptide.

Plasmids were transfected into Expi293F cells (Thermo Fisher Scientific Scientific, Waltham, MA, USA) using Expifectamine according to manufacturer’s instructions. At day 5 post-transfection, supernatant was harvested, adjusted to pH 8.0, and sterile filtered. CD52-Fc and control Fc were then purified using high-density cobalt agarose beads (GoldBio, St. Louis, MO, USA) and concentrated to 2mg/ml using Amicon Centrifugal Filters (Millipore Sigma, Burlington, MA, USA). Commercially available CD52-Fc was obtained from Arco Biosystems (Newark, DE, USA).

### Calcium Flux Measurement

B cells suspended at 10^6^ cells per ml were labeled with Indo-1 (Thermo Fisher Scientific Scientific, Waltham, MA, USA) according to the manufacturer’s instructions. After labeling and quenching, cells were resuspended in 400 µL of B cell media and incubated at 37°C for 5 min before analysis. Baseline calcium levels were recorded for 30 seconds prior to stimulation with 25 µg/ml goat anti-IgM F(ab)_2_ (Jackson ImmunoResearch, West Grove, PA, USA). Indo-1 fluorescence was detected from the UV laser of a Fortessa II (BD, San Jose, CA, USA) in DAPI and BUV396 channels. The ratiometric mean of DAPI to BUV396 at various timepoints was determined using Flowjo (version 10.5, Treestar, Ashland, OR) and was used to assess calcium flux.

### BCR-Associated Protein Kinase Phosphorylation Kinetics Using Phospho-Flow

Purified B cells were pre-incubated for 15 min with vehicle control, control Fc (20 µg/ml), CD52-Fc (20 µg/ml) in 100 µL per replicate prior to stimulation with 100 µL of anti-IgM (final concentration 25 µg/ml) for 2, 5 and 10 min with unstimulated cells serving as a baseline. In some conditions, cells were pre-treated with anti-Siglec-10 blocking antibodies (Cat. AF2130 50 µg/ml; R&D Biosystems, Minneapolis, MN, USA) (18). Cells were immediately fixed at indicated timepoints with equal volume (200 µL) of 4% paraformaldehyde in PBS. After fixation for 15 min at 37°C, cells were washed and resuspended in True-phos Perm Buffer (Biolegend, San Diego, CA, USA) for 1 h at -20°C. Subsequently, cells were washed and resuspended in antibodies against phospho-ERK, phospho-SYK, phospho-PLCγ2, phospho-BTK or phospho-Lyn (**Supplementary Table 2**) for 30 min in the dark at RT (BD Biosciences, San Jose, USA). After washing, cells were resuspended in PBS supplemented with 2% FBS and analyzed on an LSR II (BD, San Jose, CA, USA).

### Western Blot for Soluble CD52

For each condition, 10^6^ purified B cells were incubated in 200 µL of B cell media with 0 or 10 µg/ml of goat anti-IgM F(ab)_2_ (Jackson ImmunoResearch, West Grove, PA, USA) for 5 days. Cells were spun at 1200 x g for 2 min prior to collection of cell culture supernatants. The cell pellet was washed and stained with FVS510, anti-CD19 and anti-CD52 for flow cytometric analysis of surface CD52 expression at end of treatment. The supernatant was diluted 3:1 with 4x laemmli sample buffer to which β-mercaptoethanol was freshly added. Samples were boiled at 100°C for 5 min and then 45 µL from each sample loaded into wells of a 4–12% Bis Tris pre-cast gel (Bio-rad, Hercules, CA, USA). After running for 1 h at 150V, the proteins from the gel were transferred to a methanol-activated PVDF membrane using the Trans-Blot Turbo Transfer System according to manufacturer’s protocols. The membrane was blocked with 5% Milk in TBST and incubated with anti-CD52 (Clone: H186; Santa Cruz Biotechnology, Dallas, TX, USA) at 1:200 dilution overnight at 4°C. The membrane was washed 3 times for 5 min with 50 ml of TBST per wash. The membrane was then incubated with mIgGκ-HRP binding protein (Santa Cruz Biotech, Dallas, Texas, USA) at 1:1000 dilution for 1 h at RT. After washing, the membrane was developed with Femto Super Signal West (Thermo Fisher Scientific) and imaged using a c600 imager (Azure Biosystems, Dublin, CA, USA).

### RNA-Seq

Purified B cells at 10^6^ cells in 1 ml B cell media per replicate were incubated with PBS (vehicle control) or CD52-Fc for 6 h, washed and pelleted, followed by RNA extraction using the Qiagen RNEasy Mini Kit (Qiagen, Hilden, Germany) column centrifugation as per manufacturer’s protocol. Sample concentration and quality was assessed using Nanodrop. Samples were stored at -80°C and shipped to BGI genomics (San Jose, CA, USA) for library preparation and sequencing. Paired-end 100bp sequencing was performed using BGISEQ-500. Raw reads were quality checked using FASTQC, aligned using STAR and counted using featureCounts. Raw counts were normalized using the DESeq2 package in R which was used to identify differentially expressed genes between cells treated with CD52-Fc and vehicle control. An adjusted p value < 0.05 was used as a cutoff. Data is available at accession GSE163123.

### *In vitro* Stimulations

1–2 × 10^5^ healthy donor B cells isolated from whole blood buffy coats were resuspended in 100–200 µL B cell media per replicate in round bottom 96-well TC-treated plates (Greiner Bio-one, Kremsmünster, Austria) and incubated with vehicle control PBS, control Fc (20 µg/ml) or CD52-Fc (20 µg/ml) at 37°C for indicated timepoints. Where indicated, B cells were stimulated with polyclonal goat anti-IgM F(ab)_2_ (Jackson ImmunoResearch, West Grove, PA, USA) or IL-4 (20 ng/ml) (Peprotech, Cranbury, NJ, USA).

### PNGase F Treatment

5 µg of CD52-Fc or control Fc was denatured prior to the addition of PNGase F (NEB, Ipswich, MA, USA) per manufacturer’s protocol. 1 µg of protein was mixed with 4x Laemmli buffer supplemented with β-mercaptoethanol and loaded into a 4–12% Bis-Tris gel run for 1 h at 150 V. The gel was subsequently stained with Instant Blue Coomassie stain (Abcam, Cambridge, UK) and imaged on a c600 imager (Azure Biosystems, Dublin, CA, USA).

### Measuring CD52-B Cell Interaction

10 µg of CD52-Fc was pre-complexed with equimolar (~30 µg) Alexa fluor 647 anti-IgG (H+L) polyclonal antibody for 1 h at 4°C. For each condition, 10^6^ freshly isolated B cells were resuspended in 1 ml of B cell media to which either the pre-complexed CD52-Fc mixture (10 µg/ml) or secondary alone (30 µg/ml) was added and incubated for 1 h at 4°C, then washed. Fluorescence was analyzed using an LSR II (BD, San Jose, CA, USA).

### Immunofluorescence

Approximately 10^6^ isolated human B cells in suspension were fixed with 4% PFA for 10 min at room temperature then washed with PBS three times prior to blocking with 1% BSA in PBS for 1 h at RT, then resuspended in antibodies against CD52 (1:50 dilution; Clone: H186, Santa Cruz Biotech, Dallas, TX, USA) and Siglec-10 (1:40 dilution; Millipore Sigma, Burlington, MA, USA). The cells were washed three times with PBS and resuspended in blocking buffer containing Cy5 goat anti-rabbit IgG (4 µg/ml;Thermo Fisher Scientific, Waltham, MA, USA) and AlexaFluor 488^®^ donkey anti-mouse IgG (1:100 dilution; Jackson ImmunoResearch, West Grove, PA USA) secondary antibodies. Finally cells were washed three times in PBS-T, pipetted to a microscope slide and mounted with ProLong™ Gold Antifade Mountant with DAPI (Thermo Fisher Scientific, Waltham, MA, USA). Cells incubated with secondary antibodies alone were used as controls for specific staining. Slides were imaged using Zeiss 880 LSM confocal microscope.

### Data Analysis and Statistics

All flow cytometry data were analyzed with Flowjo (version 10.5, Treestar, Ashland, OR, USA). All statistical analysis was conducted using GraphPad Prism (Version 8.4.1, GraphPad Software, La Jolla, CA, USA). For non-normally distributed measurements, Mann Whitney U test was used to examine the differences between two groups. Spearman’s correlation was used to assess correlations between variables as indicated in the figure legends. For *in vitro* data Gaussian distribution was assumed and an unpaired *t*-test was applied for the comparison of two groups. For tests comparing three groups, one-way analysis of variance (ANOVA) test was used with Dunnett’s test for multiple comparisons.

## Results

### B Cell CD52 Gene Expression and Surface Glycoprotein Expression Is Elevated in SLE Patients

**Figure 1:**
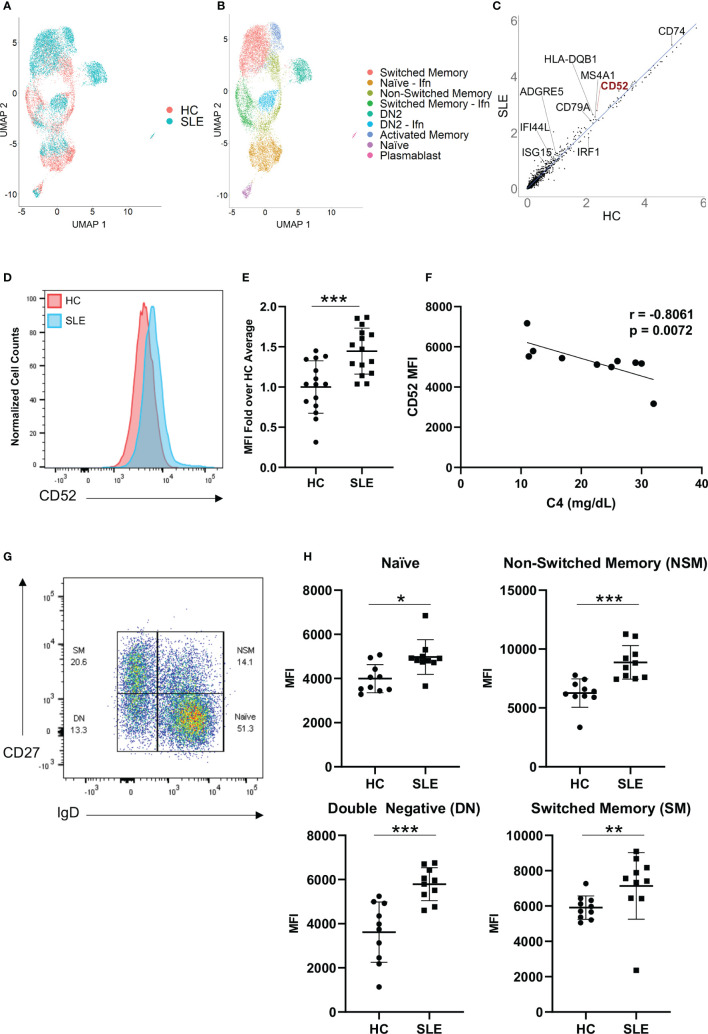
CD52 surface expression is elevated on B cells of SLE patients. **(A, B)** Single-cell RNA sequencing of HC (n=2) and SLE (n=3), represented as UMAP plots. **(A)** UMAP projections distinguishing HC vs. SLE B cells, **(B)** UMAP displaying annotated B cell subsets. Naïve – Ifn: naïve with interferon signature, Switched Memory – Ifn: switched memory with interferon signature, DN2: double-negative 2, DN2 – Ifn: double-negative two with interferon signature **(C)** Gene expression across all B cells in HC vs. SLE, scatter plot displays averaged normalized and log transformed gene expression values with select genes annotated. **(D, E)** Comparison of B cell CD52 expression between HC and SLE. **(D)** Representative flow histogram displaying surface CD52 expression on total B cells from one HC (red) and one SLE patient (blue). **(E)** CD52 expression on B cells of HC (n = 15) and SLE (n = 15) patients. Fold-increase of median fluorescence intensity (MFI) is shown, data points for each patient with mean and SD of each group. Data was pooled from two experiments. Statistics according to two-tailed Mann-Whitney U Test. **(F)** Correlation between C4 levels in SLE patients (n = 10) and corresponding CD52 surface expression (MFI). Correlations performed using Spearman’s non-parametric two-tailed method. **(G, H)** Flow cytometry assessment of CD52 expression levels on B cells. **(G)** Gating strategy for B cell subsets, pre-gated on live CD19+ cells: Naïve B cells (Naïve, IgD+CD27-), double-negative B Cells (DN, IgD-CD27-), Non-switched Memory B Cells (NSM, IgD+CD27+) and Switched Memory B cells (SM, IgD-CD27+). **(H)** Subset specific surface CD52 expression in HC (n = 10) and SLE (n = 10) with differences between groups determined by two-tailed Mann-Whitney test. *p < 0.05, **p < 0.01, ***p < 0.001.

To identify genes that are differentially regulated between HC and SLE individuals, we performed single cell RNA-sequencing on B cells from two healthy donors and three SLE patients and compared their gene expression profiles (**Figures 1A–C** and **Figure S1A**). As previously described, we found a significant expansion of the ITGAX (CD11c) and TBX21 (T-bet) expressing double negative switched memory B cell subset (DN2) in SLE patients that exhibited a strong interferon signature (**Figure 1A**) (29). In addition, multiple B cell clusters from SLE patients exhibited significantly increased expression of interferon-stimulated genes including IFI44L and ISG15 (**Figure 1C**), which was previously demonstrated to be a hallmark of SLE (30). When comparing gene expression between HC and SLE B cells across clusters, a variety of genes encoding surface proteins including CD74, HLA-DR and ADGRE5 exhibited differential expression (**Figure 1C**). Among those, CD52 was one of the most significantly upregulated genes (**Figure 1C**). To extend our findings, we used flow cytometry to analyze an independent set of SLE (n = 15) and HC (n = 15) samples for surface expression of CD52 and the other candidate genes, and to assess whether protein expression was altered in B cells derived from SLE as compared to HC patients (**Figures 1D, E**). CD52 was found to be among the most consistently upregulated surface proteins on B cells derived from SLE patients, including in B cells representing the range of B cell subsets (**Figures 1G, H**). Notably, CD52 expression was highest in B cells compared to T cells and monocytes, and was highest in the non-switched memory B cell population, which was the most depleted population in SLE patients as has been previously reported (31) (**Figures S2A, B, E**). Moreover, expression patterns of Siglec-10 across subsets corresponded to expression levels of CD52 (**Figures S2C, D**).

Surface expression of CD52 negatively correlated with complement 4 (C4) in SLE patients, (r = -0.8061, p = 0.0072), a laboratory measure of disease activity, suggesting that patients with more significant complement consumption, and therefore likely higher disease activity levels, expressed higher levels of CD52 on the surface of their B cells (**Figure 1F**).

### Soluble CD52 Is Elevated in SLE Plasma and Correlates With Clinical Parameters

**Figure 2:**
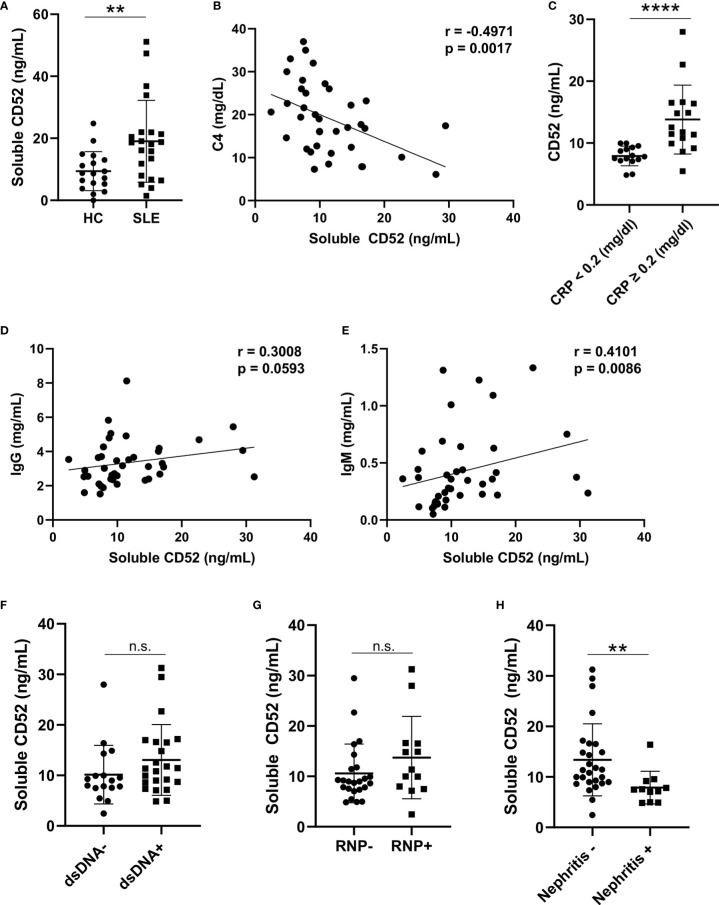
Soluble CD52 levels are elevated in SLE plasma and correlate with disease severity. **(A)** Plasma CD52 concentrations in HC (n = 18) and SLE patients (n = 22) as determined by ELISA. Differences between groups determined by two-tailed Mann-Whitney test. **(B)** Correlation between plasma CD52 and C4 levels in SLE (n = 37). Correlations were performed using Spearman’s two-tailed method. **(C)** Plasma CD52 levels in SLE patients with trace levels of C-reactive protein (<0.2 mg/dl; n = 15) or detectable levels (≥0.2 mg/dl; n = 16). Differences between groups determined by two-tailed Mann-Whitney test. **(D, E)** Correlations between plasma CD52 and **(D)** plasma IgG and **(E)** IgM levels among SLE patients (n = 40). Correlations performed using Spearman’s non-parametric two-tailed method. **(F)** Comparison of plasma CD52 levels in patients without any history of anti-dsDNA (n = 16) antibodies to those with a history of anti-dsDNA antibodies (n = 20). **(G)** Comparison of plasma CD52 levels in patients without a history of anti-RNP antibodies (n = 24) to patients with a history of anti-RNP antibodies (n = 13). **(H)** Comparison of plasma CD52 levels in patients with without any history of nephritis (n = 27) to those with active or previous diagnosis of lupus nephritis (n = 11). Two-tailed Mann-Whitney test used to compare differences between the groups. **p < 0.01, ****p < 0.0001. ns, not significant.

We next quantified levels of soluble CD52 in plasma samples from a total of 18 HC and 40 SLE patients *via* ELISA. Like cell-bound CD52, soluble CD52 was significantly elevated in SLE plasma compared to HC plasma (median [IQR] of HC vs. SLE: 8.69 [5.27, 13.01] vs. 18.36 [7.98, 21.93] ng/ml; p = 0.0074)(**Figure 2A**). We found a significant negative correlation between soluble CD52 and complement levels in SLE patients (n = 37; C3: r = -0.23, p = 0.167; C4: r = -0.50, p = 0.0017) (**Figure 2B** and **Figure S3A**). This finding suggests an association between higher levels of soluble CD52 and complement consumption in SLE patients. Furthermore, soluble CD52 was significantly elevated in SLE patients with detectable levels of C-Reactive Protein (≥ 0.2 mg/dl), an inflammatory marker, compared to those with trace amounts (< 0.2 mg/dl) (median [IQR] of trace vs. detectable: 7.805 [7.165, 9.295] vs. 12.16 [10.13, 16.47] ng/ml; p = <0.0001) (**Figure 2C**). In agreement with the correlation of soluble CD52 with measures of disease severity, we also found a weakly positive correlation of CD52 with erythrocyte sedimentation rate (ESR), another inflammatory marker (n = 32; r = 0.234, p = 0.198) (**Figure S3B**). Additionally, plasma CD52 positively correlated with IgM titers (n = 40; r = 0.41, p = 0.0086) and weakly correlated with IgG titers (n = 40; r = 0.30, p = 0.059), which were found to be significantly elevated in SLE patients (n = 20) compared to healthy controls (n = 18) (**Figures 2D, E** and **Figure S3C**). This elevation seemed broad and not primarily driven by antibodies against known lupus antigens; however we detected non-significant trends of elevated CD52 in patients with anti-dsDNA antibodies (p = 0.141) and anti-RNP antibodies (p = 0.169) (**Figures 2F, G**). Lastly, patients presenting with lupus nephritis, or who had a history of lupus nephritis, had significantly lower levels of soluble CD52 in their plasma (p = 0.0015) suggesting differences in these patients’ regulation of CD52 expression (**Figure 2H**). Correspondingly, we found a weak negative correlation between urine protein levels and plasma levels of soluble CD52 suggesting, as was observed with the nephritis cases, that patients with perturbed kidney function had deficient CD52 expression (n = 32; r = -0.213, p = 0.2421) (**Figure S3D**).

Although soluble CD52 did not correlate with SLEDAI, high CD52 levels corresponded with laboratory markers of inflammation and B cell activation with enhanced antibody production (**Figure S3E**).

### Surface CD52 Inhibits Response to B Cell Receptor Signaling

**Figure 3:**
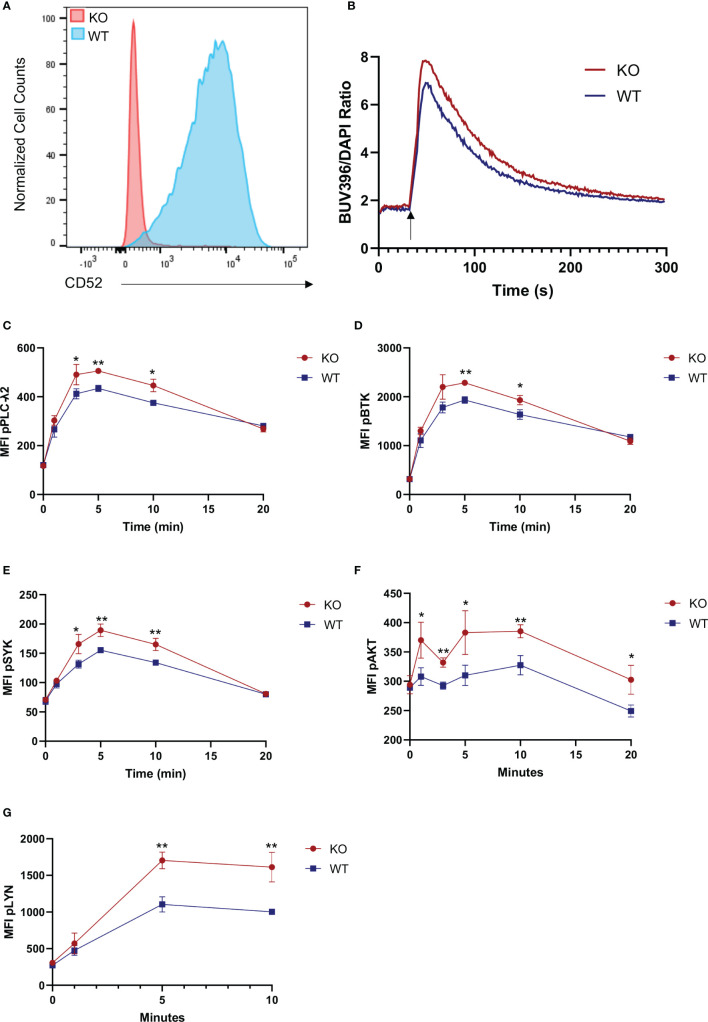
Surface CD52 expression inhibits B cell receptor signaling. **(A)** Flow cytometry data, showing a representative histogram of CD52 expression on wild-type process control JeKo-1 cells and CD52 CRISPR knockout cells. **(B)** Calcium flux measurement in CD52-KO and WT cells before and after stimulation with anti-IgM (25 µg/ml)(indicated time points). **(C–G)** Phospho-kinetics of **(C)** phospho-PLCγ2, **(D)** phospho-BTK, **(E)** phospho-SYK, **(F)** phospho-AKT and **(G)** phospho-Lyn in WT and KO cells stimulated with anti-IgM (10 µg/ml). Data in **(B, C–G)** representative of at least n = 2 independent experiments. *p < 0.05, **p < 0.01, according to unpaired t-test.

To delineate the role of surface bound CD52, we used CRISPR-Cas9 to generate CD52 knockout JeKo-1 cells, a mantle cell lymphoma cell line with high and homogenous expression of CD52 which is in contrast to expression levels in other commonly used B cell lines [(32), p. 52; (33), p. 52] (**Figure 3A**). Expression of key B cell proteins including CD19, IgM and HLA-DR were unaffected by the knockout (**Figures S4A–D**). To assess the strength of BCR signaling, we labeled wild-type (WT) and CD52-KO JeKo-1 cells with Indo-1 and measured intracellular calcium flux upon stimulation with anti-IgM. A significantly higher calcium flux response was detected in CD52-KO compared to WT cells (**Figure 3B**). To address the effect of CD52 on BCR-signaling in more depth, we measured phosphorylation levels of signaling molecules downstream of the BCR, including phospho-BTK, phospho-AKT, phospho-SYK, phospho-PLC-γ2 and phospho-Lyn following BCR engagement. We found significantly increased levels of each phospho-protein in the CD52 KO cells compared to WT cells (**Figures 3C–G**). Together, these data suggest an inhibitory role for the surface form of CD52, directly or indirectly affecting the BCR signaling pathway.

To assess the possibility of cis-interaction of CD52 with inhibitory receptor Siglec-10, we immunofluorescently stained human B cells for CD52 and Siglec-10 to assess the extent of co-localization and found that both proteins were broadly distributed across the B cell surface and partially co-localized, suggesting a baseline level of cis-interaction (**Figure S5A**).

### CD52 Gene Expression Correlates With MHC Class II Gene Expression in B Cells and Is Lowest in Affinity Maturing B Cells

In order to gain further insights into the function of surface CD52 in B cells on a broader scale, we assessed expression of CD52 in publicly available single-cell RNA-seq datasets from PBMC samples of healthy individuals (10X Genomics). In correlating the expression values of CD52 with all detected genes within each individual B cell, we found MHC Class II proteins (HLA-DRA, CD74) and associated tetraspanins (CD37, CD81, CD53) to be among the most consistently positively correlated genes across multiple independent datasets (**Figure S6A**).

In addition, we analyzed publicly available single-cell RNA-seq data (accession number: E-MTAB-9005) from human tonsil tissue to examine subset specific analysis of gene expression along the B cell differentiation trajectory (**Figure S6B**). Naïve B cells expressed relatively high levels of CD52, and expression decreased upon activation. CD52 expression continued to decrease along the differentiation trajectory as B cells entering the germinal center (Pre-GC: pre-germinal center, DZ: dark zone) expressed lower levels than activated naïve cells. Within the germinal center, lowest levels were expressed among cycling and light zone (LZ) B cells which interact with T cells and dendritic cells to undergo affinity maturation (34). Antibody secreting plasmablasts, arising post-GC, had the lowest expression of CD52 across all subsets while memory B cells had the highest. In summary, peripheral B cell subsets including naïve and memory B cells, which require tight regulation of BCR signaling for maintenance of tolerance, exhibited the highest expression of CD52, whereas B cell subsets undergoing differentiation or having effector functions exhibited the lowest levels of expression.

### CD52 Is Cleaved on Stimulated B Cells *via* PLC

**Figure 4:**
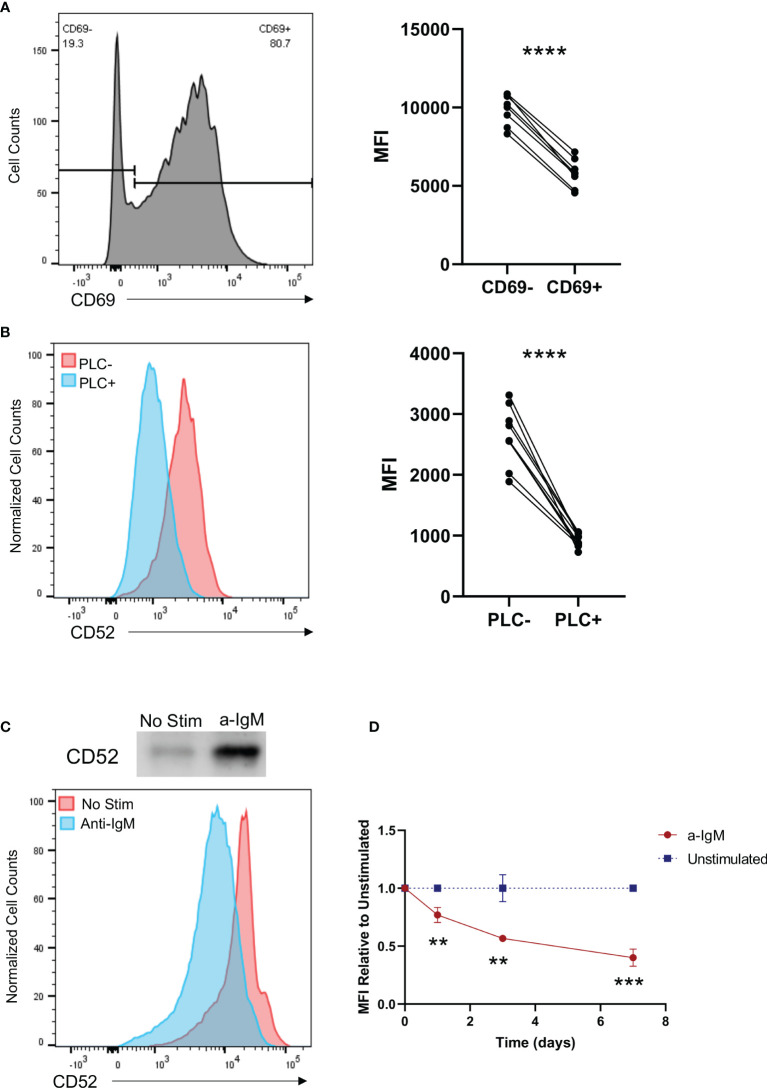
Activation of B cells results in cleavage of CD52 from their cell surface in a phospholipase C dependent manner. **(A)** Purified B cells were stimulated with anti-IgM (10 µg/ml) and IL-4 (20 ng/ml) for 40 h prior to staining. Paired CD52 MFI shown between CD69- and CD69+ cells within each replicate, gated on live CD19+ cells. Paired t-test was used to compare the two groups. **(B)** HC (n = 5) and SLE (n = 5) PBMCs were treated with PLC and stained for expression of CD52. Paired surface CD52 expression (MFI) shown for treated and untreated cells gated on live CD19+ cells. Paired t-test was used to compare the two groups. **(C)** Western blot of supernatant CD52 shown with corresponding histogram displaying surface CD52 expression of cells in respective conditions. **(D)** Purified B cells were left unstimulated or stimulated with anti-IgM (2.5 µg/ml) and stained at indicated time points for surface CD52. CD52 expression among stimulated cells is shown relative to MFI of unstimulated cells at each time point, gated on live CD19+ cells. Differences between the groups were calculated with unpaired two-tailed t-test. Data in **(A–D)** representative of at least n = 2 independent experiments. **p < 0.01, ***p < 0.001, ****p < 0.0001. ns, not significant.

To understand the relationship between B cell activation and CD52 surface expression, we analyzed B cells derived from healthy donors stimulated for 40 h with a combination of the B cell activating factors anti-IgM and IL-4. We used the marker CD69 to delineate activated cells and found that CD52 surface expression was significantly lower in CD69 positive cells (**Figure 4A**).

Given that BCR stimulation induces activation of PLC, which cleaves GPI anchors, we assessed the ability of PLC to cleave CD52 by treating PBMCs from HC (n = 5) and SLE (n = 5) donors with the enzyme, and analyzed CD52 surface expression on treated and untreated cell populations (35). As expected, CD52 surface expression was significantly diminished in PLC treated populations, with no differences in the ability of PLC to cleave CD52 on HC vs. SLE PBMCs (**Figure 4B** and **Figure S7A**). Notably, the effect of PLC on surface levels of CD52 was more pronounced in T cells and monocytes than in B cells, indicating that CD52 on B cells might be protected from PLC mediated cleavage and that differential mechanisms are involved in CD52 cleavage from different cell types (36) (**Figures S7A, B**).

To characterize the ability of B cells to cleave and generate soluble CD52, we measured CD52 in supernatants of isolated healthy donor B cells *via* western blot 5 days after IgM stimulation. We used flow cytometry to analyze corresponding cell surface CD52 expression. Soluble CD52 levels were significantly elevated in the supernatants of stimulated B cells, while corresponding cell surface expression decreased upon IgM stimulation, indicating cleavage of CD52 from the surface of stimulated cells as a significant mechanism for the lower surface CD52 expression on activated cells (**Figure 4C**). Cleavage or downregulation of CD52 from the cell surface was also time-dependent; cells continuously stimulated with anti-IgM showed lower surface expression on days 3 and 7 compared to 24 h post-stimulation (**Figure 4D**).

### Soluble CD52 Inhibits B Cell Receptor-Mediated Signaling

To elucidate the role of soluble CD52 in B cell function, we recombinantly expressed CD52 protein as a dimeric Fc fusion protein, along with the corresponding Fc control (**Figure S8A**). The proteins were engineered with the LALA-PG mutations in the Fc region to minimize binding to Fc receptors on B cells (27, 28). We confirmed the presence of a large (~8 kDa) glycan by comparing the molecular weight of PNGase F treated and untreated protein (**Figure S8B**). Additionally, we used a MAL-II coated ELISA to confirm the presence of α-2,3 sialylation which has been reported as the glycan moiety that confers the most bioactivity (37) (**Figure S8C**). Using flow cytometry, we confirmed binding of CD52-Fc to B cells by comparing fluorescent intensities of CD52-Fc pre-complexed with AlexaFluor 647 anti-IgG antibody to secondary antibody alone (**Figure S8D**).

**Figure 5:**
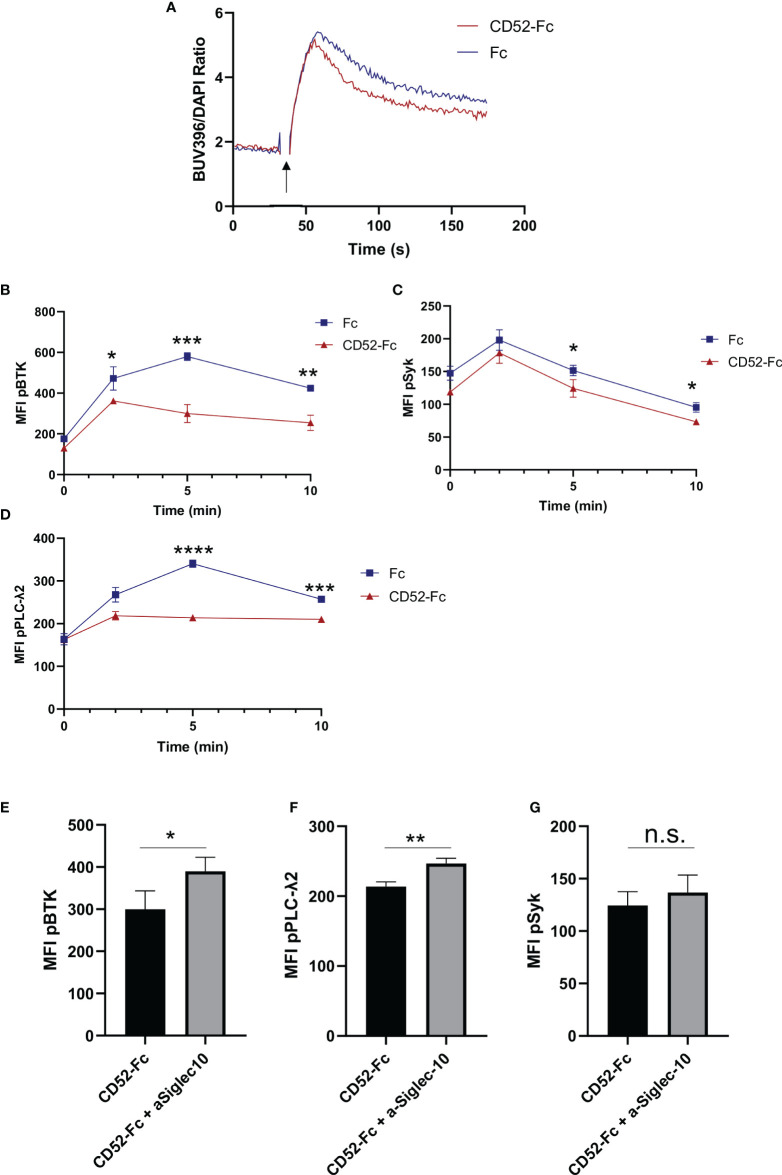
Soluble CD52 inhibits B cell receptor signaling. **(A)** Calcium flux measurement in human B cells pre-incubated with either Fc (20 µg/ml) or CD52-Fc (20 µg/ml) prior stimulation with anti-IgM (25 µg/ml) (indicated timepoints). **(B–D)** Phospho-kinetics of **(B)** phospho-BTK, **(C)** phospho-SYK, and **(D)** phospho-PLCγ2 stimulated with anti-IgM (25 µg/ml) for B cells pre-incubated either Fc (20 µg/ml) or CD52-Fc (20 µg/ml). **(E–G)** Phospho-protein comparison of BTK, PLCγ2 and Syk, 5 min post-stimulation of cells pre-treated with anti-Siglec10 blocking antibody (50 µg/ml) and CD52-Fc (20 µg/ml) or CD52-Fc alone. Unpaired two-tailed t-test used to compare groups. Data in **(A–G)** representative of at least n = 3 independent experiments. *p < 0.05, **p < 0.01, ***p < 0.001, ****p < 0.0001.

Given the observed effect of cell-bound CD52 on BCR signaling (**Figures 3C–F**), as well as the reported role of soluble CD52 in inhibiting T cell receptor signaling (18), we evaluated whether soluble CD52 inhibited BCR mediated signaling. B cells were incubated for 15 min with control Fc, or CD52-Fc prior to stimulation with anti-IgM (25 µg/ml). We measured the impact of control Fc or CD52-Fc pre-incubation on calcium flux responses using Indo-1 labeled B cells and found that CD52-Fc significantly diminished calcium flux responses over the Fc control (**Figure 5A**). To further investigate the impact of CD52-Fc on BCR signaling we assessed phosphorylation of signaling molecules downstream of the BCR at serial timepoints by flow cytometry. In accordance with the calcium flux data and similar to the effect of cell-bound CD52, soluble CD52 significantly diminished BCR responses, with lower levels of phospho-BTK, phospho-SYK, and phospho-PLC-γ2 at various timepoints (**Figures 5B–D**). To test if the observed effect was mediated by Siglec-10, we blocked extracellular Siglec-10 prior to incubation of B cells with CD52-Fc and observed partially reversed inhibitory effects of CD52-Fc (**Figures 5E–G**).

### Soluble CD52 Reduces Expression of Surface Immunoglobulin

**Figure 6:**
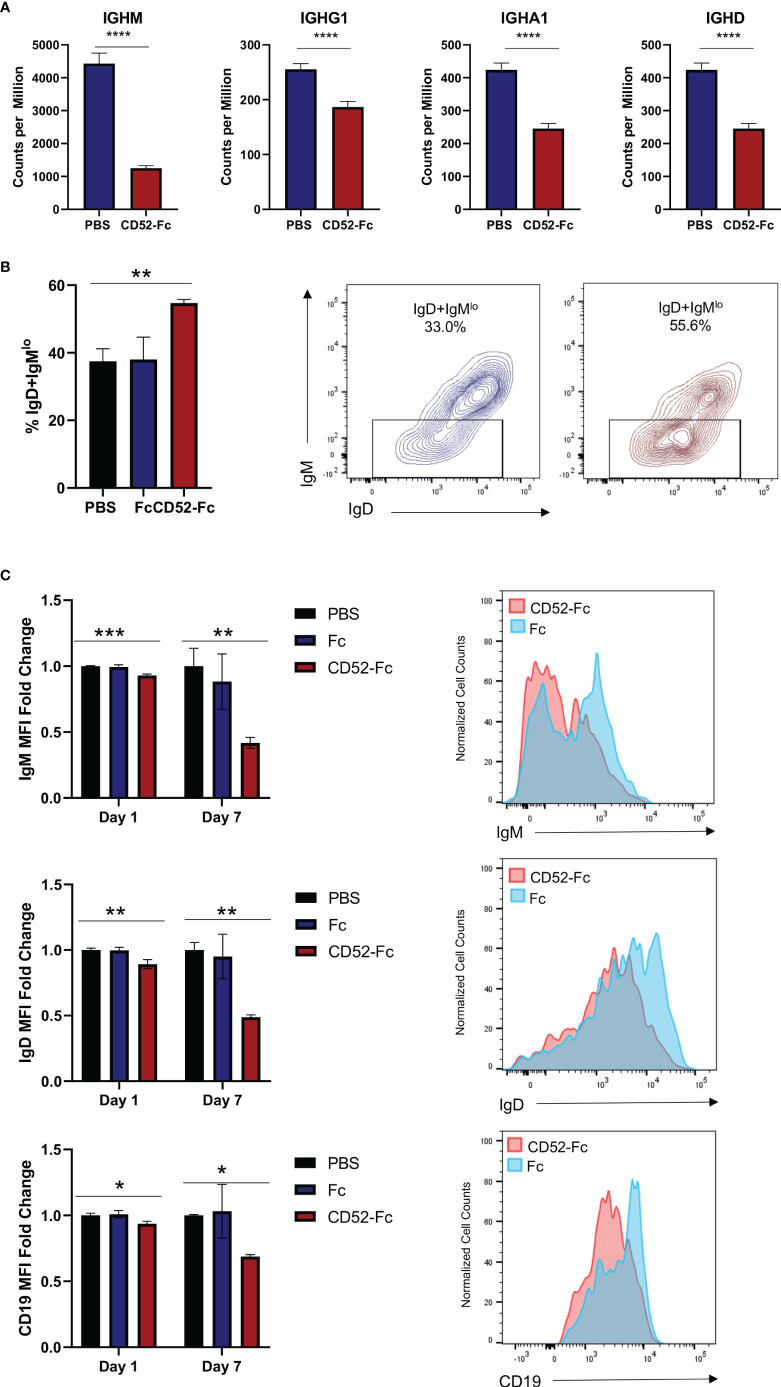
Soluble CD52 reduces expression of surface immunoglobulin. **(A)** Bar plot of gene expression levels for the indicate gene. Data represent mean ± SD, * indicates DEG between CD52-Fc treated and vehicle control (Padj < 0.05) as determined by deseq2. **(B)** Healthy donor B cells were cultured for 7 days with vehicle control, Fc (20 µg/ml), or CD52-Fc (20 µg/ml) and the proportion of IgD+IgM^lo^ anergic B cells among live naïve CD19+IgD+ B cells was quantified. **(C)** B cells were cultured with vehicle control, Fc (20 µg/ml), or CD52-Fc (20 µg/ml) and assessed for expression levels of surface IgM, IgD, and CD19 at indicated timepoints. Data expressed as MFI ratio relative to average vehicle control MFI. One-way analysis of variance was used in conjunction with Dunnett’s test for multiple comparisons to compare groups. Data in **(B, C)** representative of at least n = 3 independent experiments. *p < 0.05, **p < 0.01, ***p <0.001, ****p < 0.0001.

To identify changes in gene expression impacted by soluble CD52, we incubated cells with CD52-Fc or vehicle control for 6 h prior to lysing cells and extracting RNA for RNA-seq. Among the most differentially expressed genes were immunoglobulin genes as well as genes related to the B cell receptor complex including IgM, IgD and CD19 (**Figure 6A**). In agreement with this we observed a significant expansion of the previously reported IgD+IgM^lo^ anergic B cells after 7 days of incubation with CD52-Fc (38) (**Figure 6B**). To confirm whether the changes in gene expression translated to protein expression changes for immunoglobulin, we incubated B cells with PBS, control Fc, or CD52-Fc for 72 h or 7 days, after which we assessed changes in expression of surface immunoglobulin and CD19 by flow cytometry. CD52-Fc treatment significantly reduced IgM, IgD, and CD19 expression, with greater reduction in surface expression detected on day 7 (**Figure 6C**). Unlike in monocytes, incubation of high dose CD52-Fc (100 µg/ml) with B cells did not induce apoptosis (19) (**Figure S9A**).

### Soluble CD52 Reduces Surface Expression of CXCR5 on B Cells and Responses to CXCL13

In addition to changes in the expression of B cell receptor related proteins, we observed that incubation of healthy donor B cells with CD52-Fc significantly reduced surface expression of CXCR5, a chemokine receptor important for B cell homing to lymph nodes (39) (**Figures S10A, B**). Moreover, calcium flux in response to CXCL13, the ligand for CXCR5, was significantly diminished in cells pre-incubated with CD52-Fc compared to control Fc for 24 h (**Figure S10C**).

## Discussion

Alterations in B cell phenotype and activation states in SLE have been extensively described (1, 40–42). B cells from SLE patients have lower surface expression of the CD19/CD21 receptor complex but increased expression of other receptors including CXCR4 and IL-21R (9, 43, 44); a number of these differentially expressed proteins are thought to drive disease pathogenesis (45–47), while others are considered to be biomarkers that reflect immune dysregulation in these patients (48, 49). B cells in SLE are considered hyperactivated and drivers of disease pathogenesis (50). However, more recent studies have highlighted significant hyporesponsiveness among B cells derived from SLE patients [(9), p. 9; (10)]. In particular, signaling abnormalities are restricted to the B cell compartment and encompass responses to TLR9 signaling and BCR signaling, but not TLR7 signaling (9). The hyporesponsiveness is hypothesized to be a byproduct of an anergic cell state acquired by chronic antigen stimulation of B cells in the absence of secondary signals, such as those provided by cognate T cells (10). Homeostatic features, such as the hyporesponsiveness of SLE B cells and receptor editing, play a prominent role in defining the course of disease pathogenesis (10, 51, 52). Here, we used an unbiased single cell RNA sequencing approach to identify CD52, a small glycoprotein, as differentially expressed on the B cells of SLE patients, and we establish its role as an inhibitory autoregulatory protein.

We observed significantly increased CD52 gene expression and surface expression on B cells, as well as increased soluble CD52 in plasma, derived from SLE patients compared to healthy controls. Increases in B cell surface CD52 expression and plasma soluble CD52 levels correlated positively with some measures of disease activity, including reduced complement levels and increased C-reactive protein levels suggesting its potential utility as a disease biomarker. Based on our finding that CD52 is cleaved from the B cell surface upon BCR engagement, we hypothesize that the negative correlation we observed between plasma soluble CD52 levels in plasma and complement levels might reflect increases in chronic antigen stimulation in patients with more severe disease. In support of that view, plasma CD52 levels correlated positively with immunoglobulin titers, a reflection of increased antigenic engagement and plasma cell differentiation. As we show that elevated levels of cell-bound and soluble CD52 have predominantly inhibitory effects on B cell activation, we hypothesize that up-regulation of CD52 and CD52-cleavage is a negative feedback mechanism, intended to limit hyperactive B cell responses.

Our data provides evidence for an inhibitory effect of cell-bound and soluble CD52 on B cells. Using a knockout cell line generated with CRISPR, we found that B cells lacking CD52 surface expression were hyper-responsive to B cell receptor stimulation compared to wild type cells. Notably, treatment of PBMCs with phospholipase C led to complete cleavage of CD52 from T cells and monocytes, while a significant fraction of CD52 remained membrane-bound on B cells, suggesting the presence of a non-cleavable form of CD52 restricted to the B cell compartment. This is consistent with our observation that CD52 in its membrane-bound and its soluble states is elevated in SLE patients. Given our finding that both forms regulate responses to BCR signaling, CD52 may be an important inhibitory regulatory molecule in SLE. Increases in surface CD52 expression on B cells of SLE patients may in part explain the hyporesponsiveness of SLE patient-derived B cells.

Through interrogation of a wide range of publicly available single-cell RNA-seq PBMC datasets, we found that CD52 expression strongly correlates with expression of MHC class II and surface tetraspanins including CD37 and CD53, which are known to associate with MHC Class II. Though not in the scope of this study, the co-expression of CD52 with MHC class II proteins might imply cell surface associations of these proteins or involvement of CD52 in MHC class II signaling. Moreover, when looking at B cell subset-specific gene expression of CD52, we found that CD52 was most highly expressed on mature circulating cells such as memory and naïve B cells compared to germinal center (GC) B cells and plasmablasts (34). The significantly higher expression on circulating B cells, which require precise regulation of BCR signaling responses, supports a role for CD52 in peripheral tolerance.

Based on previous findings in T cells and monocytes, soluble CD52 has been established as an inhibitory molecule that exerts its effects *via* Siglec-10 and other as yet undefined receptors (18, 19). As with T cells, antigen stimulation of B cells leads to phospholipase C activation, which then cleaves CD52 from the B cell surface. Activated B cells exhibited significantly lower levels of CD52 surface expression. We hypothesize that this could be a homeostatic mechanism whereby cleavage of surface CD52 enables more complete cellular responses, while the soluble form of CD52 can act in an autocrine or paracrine manner to regulate local B cell responses to antigen receptor signaling. Given the detectable levels of CD52 observed in the supernatant of stimulated cells, we generated recombinant CD52-Fc to elucidate the role of soluble CD52. We found that CD52-Fc significantly diminished responses to BCR signaling, and blockade of Siglec-10 partially reversed the inhibition, suggesting Siglec-10 as a potential receptor for soluble CD52 on B cells. However, as our observed effect of blockade of Siglec-10, which is known to be widely expressed on B cells, is incomplete, it suggests that other as yet undefined receptors for CD52 may contribute to its effects on B cells. As has been reported, α2,3 sialylation of the glycan accounts for most of the inhibitory effects, and a wide range of receptors such as CD22 bind sialic acids (53). CD24 is another highly sialylated GPI-linked protein expressed on transitional B cells and, as has been reported for CD52, can both sequester HMGB1 and bind Siglec-10 [(54), p. 24; (55, 56), p. 24]. Therefore, it is possible that CD52 and CD24 have redundant inhibitory functions, with CD52 playing a more prominent role in mature B cell subsets which highly express CD52 but not CD24. HMGB1 is significantly elevated in SLE patients with purported pro-inflammatory roles; therefore, the ability of soluble CD52 to sequester HMGB1 is potentially another autoregulatory function in SLE patients (57). It is important to note that there was batch to batch variation in the bioactivity of CD52-Fc produced from the same host Expi293F cells, likely due to variations in glycosylation, which has been well-described (18, 19, 37).

Lastly, in order to understand stimulation-independent effects of soluble CD52 on B cell function we incubated B cells with CD52-Fc without any stimulation to understand the influence of CD52 on B cell behavior. Notably, we found significant downregulation of surface immunoglobulin. Lower surface expression of immunoglobulin has been found to correspond with lower responsiveness to antigen stimulation (38). In particular, we found a significant expansion of IgD+IgM^lo^ anergic B cells in groups incubated with CD52-Fc, indicating the ability of CD52-Fc to partially induce anergy in B cells (38). In addition to its impact on surface immunoglobulin, we also found that CD52 decreases the surface expression of the chemokine receptor CXCR5, which recognizes the ligand CXCL13 to promote homing to secondary lymphoid organs (39). Mouse models of SLE in which CXCR5 is knocked out have shown significantly diminished disease onset, implying an important role for the receptor in disease pathogenesis [(58), p. 5]. Our findings with immunoglobulin and CXCR5 expression suggest a multi-faceted role for soluble CD52 in regulating B cells in SLE.

Another potential inhibitory role for CD52 in SLE is in reducing excessive complement activation. Previous work has shown that C1q, which recognizes immune complexes as part of the classical pathway, co-immunoprecipitates with CD52 [(15), p. 52]. Moreover, rituximab-resistant cell lines over-expressing CD52 are particularly resistant to complement-mediated cytotoxicity which is partially reversed by CD52 blockade and supports its role as a complement inhibitory protein (14). Therefore, higher plasma and B cell surface levels of CD52 in SLE patients may serve to sequester C1q, thereby limiting end organ damage that results from excessive complement activation. In support of this hypothesis, we observed significantly higher levels of soluble CD52 in patients without lupus nephritis compared to those with a history of lupus nephritis.

Also supporting an inhibitory role for CD52 is the observation that 20% and 3% of MS patients treated with lymphocyte-depleting alemtuzumab develop thyroid autoimmunity and autoimmune thrombocytopenia respectively within months or years after start of treatment. Moreover, 5.5% of alemtuzumab-treated patients develop sustained non-thyroid autoantibodies. Although the mechanism of secondary autoimmunity is not yet established, increased expression of B-cell activating factor (BAFF) in patients post-treatment, and the elevation of serum IL-21 in patients who develop autoimmunity, both of which are important factors for B cell differentiation and antibody production, support a B cell driven mechanism (59, 60). Based on these observations, we propose that both the predominance of immature B cells expressing low levels of CD52 during the rapid reconstitution of the B cell compartment following treatment, as well as the sequestration of soluble CD52 by therapeutic antibody induce a homeostatic imbalance that promotes breaks in B cell tolerance that in turn drive secondary autoimmunity (59).

This study has several limitations. For one, this study does not address how higher expression of CD52 is induced in SLE patients or the milieu of soluble factors in patient serum as well as the potential effects of immunosuppressive therapies, both of which might influence CD52 expression. Previous studies assessing CD52 expression in a cohort with relatively active disease noted the expansion of CD4+CD52^lo^ T cells and decrease in soluble CD52, particularly in untreated patients, highlighting the role that disease status and use of immunomodulatory therapies has on CD52 expression (61). The majority of SLE patients recruited as part of this study had well controlled disease, which would support the notion that increased expression of soluble CD52 in these patients might reflect a homeostatic mechanism involved in improving or limiting their disease severity. Lastly, despite extending and validating our findings in larger sets of patients, our single cell RNA-Seq findings are based on analysis of a small number of patients.

In summary, we demonstrated increased surface and plasma levels of CD52 in patients with SLE compared to HCs. Our findings suggest that CD52 serves as a homeostatic protein that exerts inhibitory effects on B cell BCR signaling, immunoglobulin expression, and chemokine receptor expression. Further studies will be needed to establish which factors induce the upregulation of surface CD52 expression, particularly in SLE patients, and to investigate the possibility of additional receptors for CD52 on B cells. The differential regulation of surface-bound and cleavable CD52 is still unknown and will be critical for advancing our understanding of the role CD52 plays in B cell homeostasis. The inhibitory effects of CD52 on B cells suggests the potential to therapeutically enhance its activity for the treatment of SLE. Future studies are needed to evaluate the potential to leverage the inhibitory activity of CD52 on B cells as a novel therapeutic strategy for SLE and other autoimmune diseases.

## Data Availability Statement

The datasets presented in this study can be found in online repositories. The name of the repository and accession numbers can be found here: NCBI, accession: GSE163121 and GSE163123.

## Ethics Statement

The studies involving human participants were reviewed and approved by Stanford Institutional Review Board. The patients/participants provided their written informed consent to participate in this study.

## Author Contributions

KB planned and performed the experiments, analyzed the data, interpreted results, and prepared the manuscript. JS generated recombinant protein for use in experiments. YC and MB helped with obtaining human samples and clinical data. TL discussed data and edited the manuscript. AZ processed raw RNA-seq data. YR aided with experiments and analysis of data. JC and WR provided input on experimental design, data analysis, and edited the manuscript. All authors contributed to the article and approved the submitted version.

## Funding

Funding was provided by NIH NIAMS R01 AR063676, NIH NIAMS UH2 AR067681, NIH NIAID U19 AI11049103, and NIH NIAID U01 AI101981 to WHR. KB received support from the National Science Foundation Graduate Research Fellowship under grant no. DGE-1656518.

## Conflict of Interest

The authors declare that the research was conducted in the absence of any commercial or financial relationships that could be construed as a potential conflict of interest.
