# Supplementary material for: CD52 Is Elevated on B cells of SLE Patients and Regulates B Cell Function
Source: Front Immunol. 2021 Feb 4;11:626820. doi: 10.3389/fimmu.2020.626820 (PMC7917337; doi:10.3389/fimmu.2020.626820)
Supplement: Supplementary file 1 [file Presentation_1.pptx]

## Slide 1
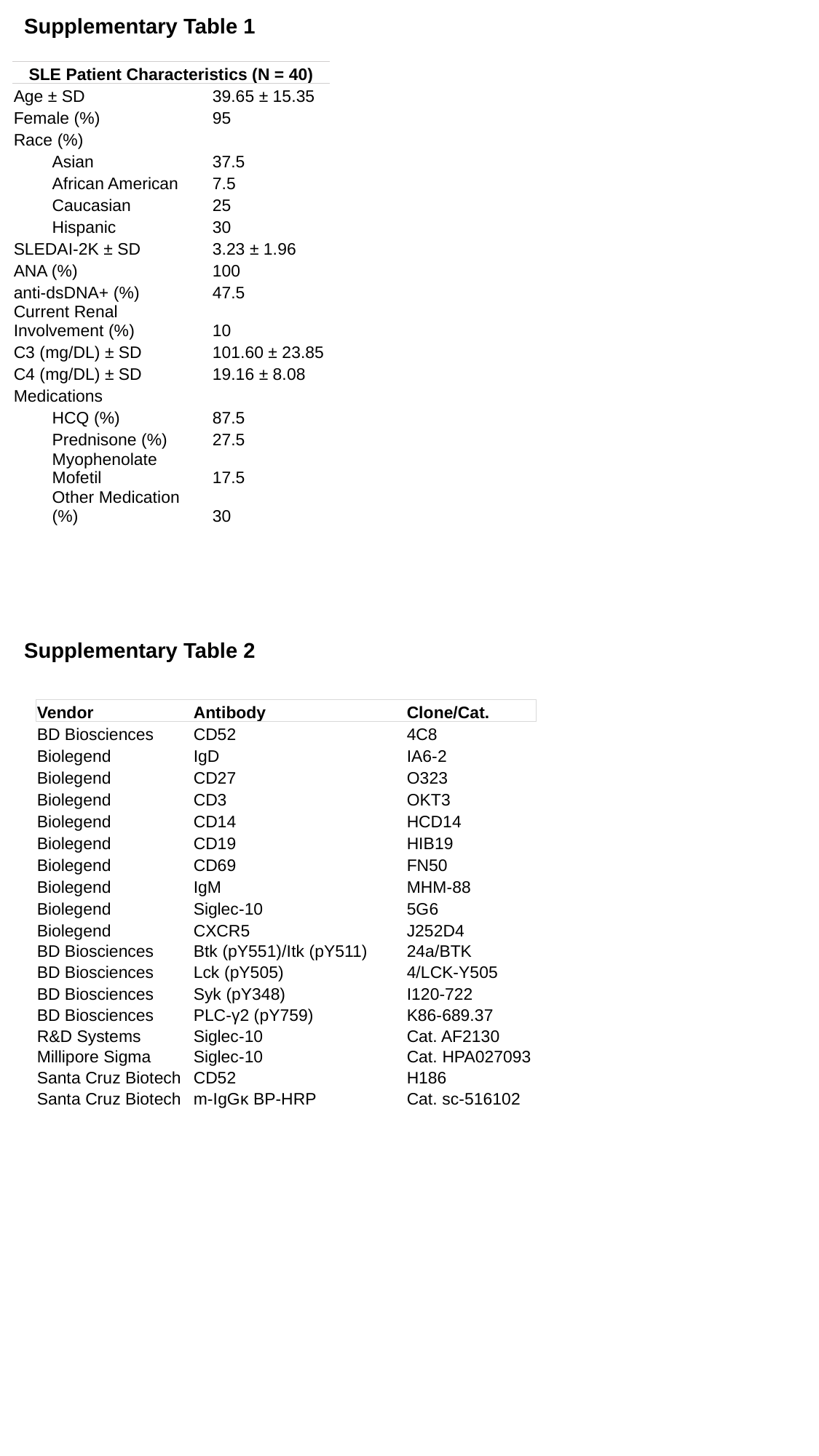

Supplementary Table 1
| SLE Patient Characteristics (N = 40) | | |
| --- | --- | --- |
| Age ± SD | | 39.65 ± 15.35 |
| Female (%) | | 95 |
| Race (%) | | |
| | Asian | 37.5 |
| | African American | 7.5 |
| | Caucasian | 25 |
| | Hispanic | 30 |
| SLEDAI-2K ± SD | | 3.23 ± 1.96 |
| ANA (%) | | 100 |
| anti-dsDNA+ (%) | | 47.5 |
| Current Renal Involvement (%) | | 10 |
| C3 (mg/DL) ± SD | | 101.60 ± 23.85 |
| C4 (mg/DL) ± SD | | 19.16 ± 8.08 |
| Medications | | |
| | HCQ (%) | 87.5 |
| | Prednisone (%) | 27.5 |
| | Myophenolate Mofetil | 17.5 |
| | Other Medication (%) | 30 |
Supplementary Table 2
| Vendor | Antibody | Clone/Cat. |
| --- | --- | --- |
| BD Biosciences | CD52 | 4C8 |
| Biolegend | IgD | IA6-2 |
| Biolegend | CD27 | O323 |
| Biolegend | CD3 | OKT3 |
| Biolegend | CD14 | HCD14 |
| Biolegend | CD19 | HIB19 |
| Biolegend | CD69 | FN50 |
| Biolegend | IgM | MHM-88 |
| Biolegend | Siglec-10 | 5G6 |
| Biolegend | CXCR5 | J252D4 |
| BD Biosciences | Btk (pY551)/Itk (pY511) | 24a/BTK |
| BD Biosciences | Lck (pY505) | 4/LCK-Y505 |
| BD Biosciences | Syk (pY348) | I120-722 |
| BD Biosciences | PLC-γ2 (pY759) | K86-689.37 |
| R&D Systems | Siglec-10 | Cat. AF2130 |
| Millipore Sigma | Siglec-10 | Cat. HPA027093 |
| Santa Cruz Biotech | CD52 | H186 |
| Santa Cruz Biotech | m-IgGκ BP-HRP | Cat. sc-516102 |

## Slide 2
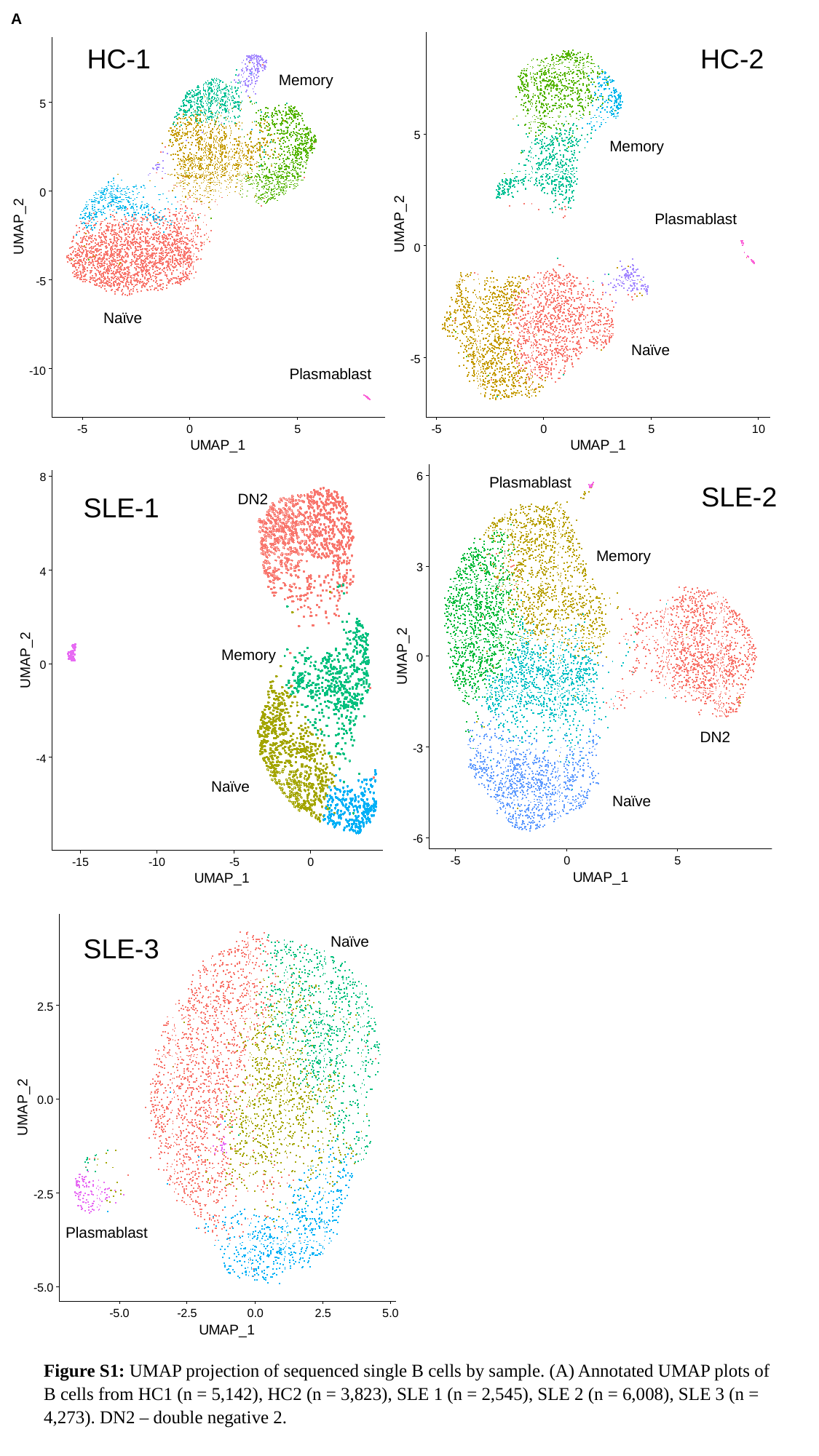

A
HC-1
HC-2
Memory
Memory
Plasmablast
Naϊve
Naϊve
Plasmablast
Plasmablast
SLE-2
DN2
SLE-1
Memory
Memory
DN2
Naϊve
Naϊve
SLE-3
Naϊve
Plasmablast
Figure S1: UMAP projection of sequenced single B cells by sample. (A) Annotated UMAP plots of B cells from HC1 (n = 5,142), HC2 (n = 3,823), SLE 1 (n = 2,545), SLE 2 (n = 6,008), SLE 3 (n = 4,273). DN2 – double negative 2.

## Slide 3
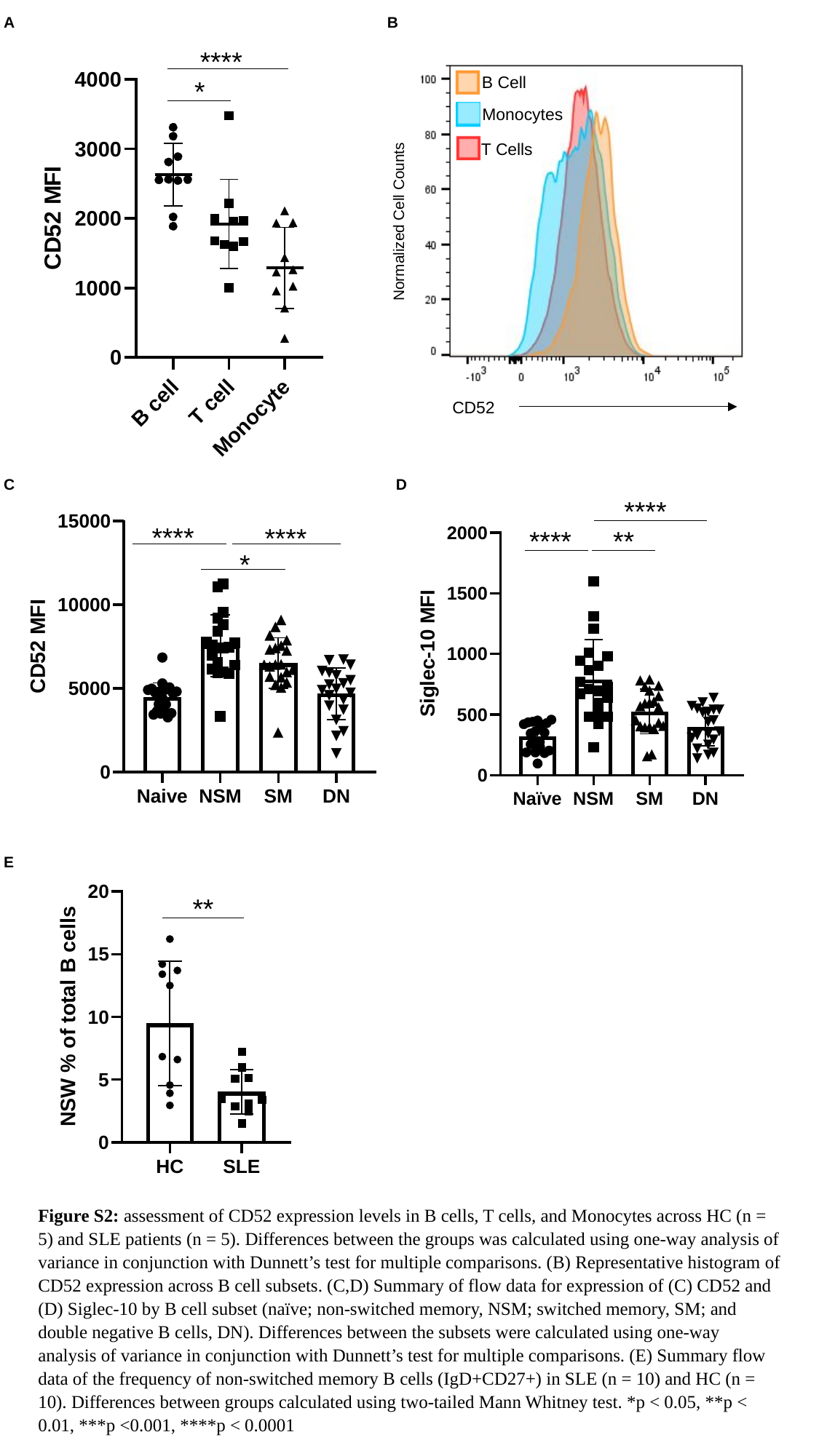

B
A
****
B Cell
*
Monocytes
T Cells
Normalized Cell Counts
CD52
C
D
****
****
****
**
****
*
E
**
Figure S2: assessment of CD52 expression levels in B cells, T cells, and Monocytes across HC (n = 5) and SLE patients (n = 5). Differences between the groups was calculated using one-way analysis of variance in conjunction with Dunnett’s test for multiple comparisons. (B) Representative histogram of CD52 expression across B cell subsets. (C,D) Summary of flow data for expression of (C) CD52 and (D) Siglec-10 by B cell subset (naïve; non-switched memory, NSM; switched memory, SM; and double negative B cells, DN). Differences between the subsets were calculated using one-way analysis of variance in conjunction with Dunnett’s test for multiple comparisons. (E) Summary flow data of the frequency of non-switched memory B cells (IgD+CD27+) in SLE (n = 10) and HC (n = 10). Differences between groups calculated using two-tailed Mann Whitney test. *p < 0.05, **p < 0.01, ***p <0.001, ****p < 0.0001

## Slide 4
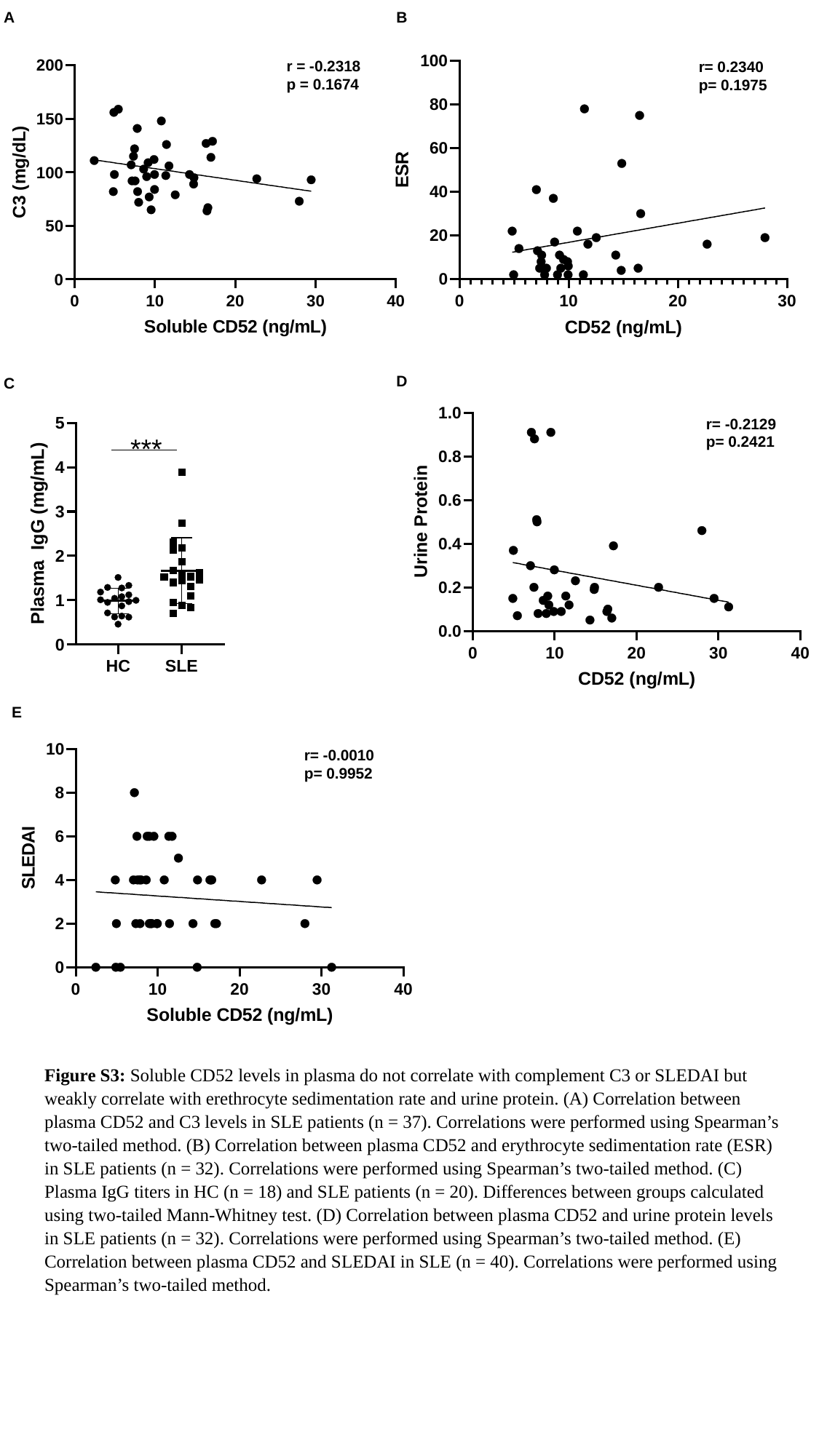

A
B
r = -0.2318
p = 0.1674
r= 0.2340
p= 0.1975
D
C
r= -0.2129
p= 0.2421
***
E
r= -0.0010
p= 0.9952
Figure S3: Soluble CD52 levels in plasma do not correlate with complement C3 or SLEDAI but weakly correlate with erethrocyte sedimentation rate and urine protein. (A) Correlation between plasma CD52 and C3 levels in SLE patients (n = 37). Correlations were performed using Spearman’s two-tailed method. (B) Correlation between plasma CD52 and erythrocyte sedimentation rate (ESR) in SLE patients (n = 32). Correlations were performed using Spearman’s two-tailed method. (C) Plasma IgG titers in HC (n = 18) and SLE patients (n = 20). Differences between groups calculated using two-tailed Mann-Whitney test. (D) Correlation between plasma CD52 and urine protein levels in SLE patients (n = 32). Correlations were performed using Spearman’s two-tailed method. (E) Correlation between plasma CD52 and SLEDAI in SLE (n = 40). Correlations were performed using Spearman’s two-tailed method.

## Slide 5
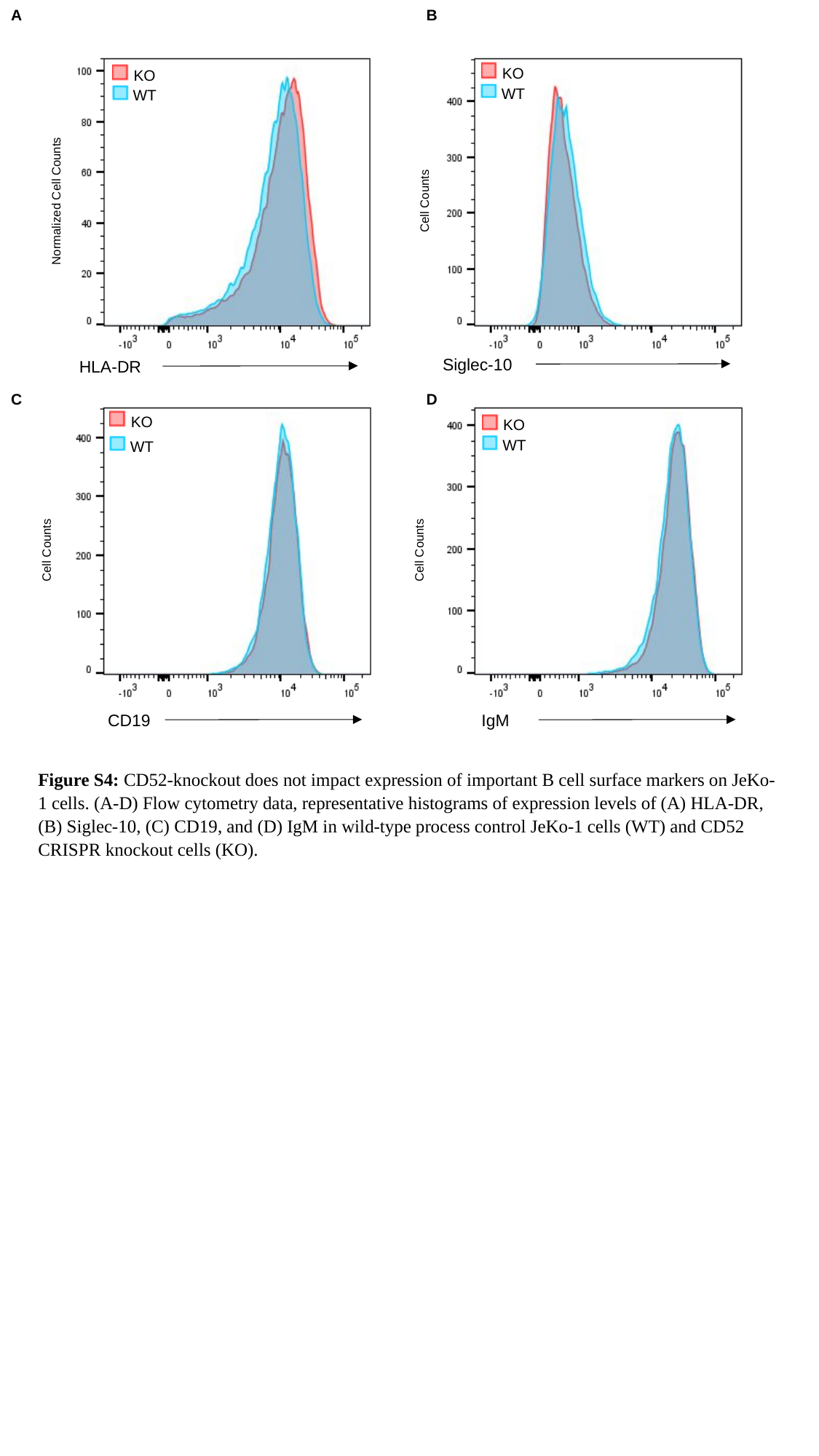

A
B
KO
KO
WT
WT
Normalized Cell Counts
Cell Counts
Siglec-10
HLA-DR
C
D
KO
KO
WT
WT
Cell Counts
Cell Counts
CD19
IgM
Figure S4: CD52-knockout does not impact expression of important B cell surface markers on JeKo-1 cells. (A-D) Flow cytometry data, representative histograms of expression levels of (A) HLA-DR, (B) Siglec-10, (C) CD19, and (D) IgM in wild-type process control JeKo-1 cells (WT) and CD52 CRISPR knockout cells (KO).

## Slide 6
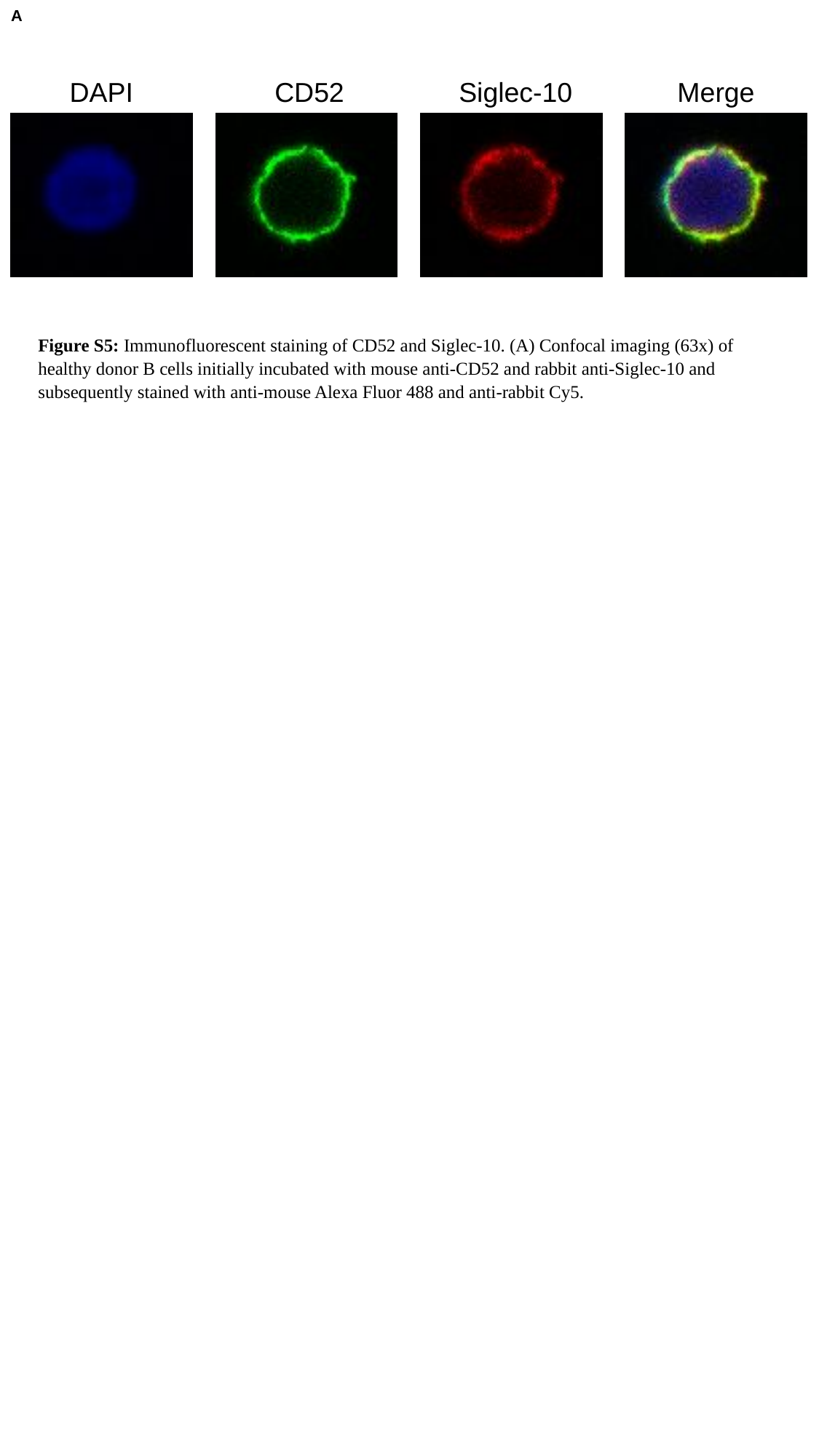

A
DAPI
CD52
Siglec-10
Merge
Figure S5: Immunofluorescent staining of CD52 and Siglec-10. (A) Confocal imaging (63x) of healthy donor B cells initially incubated with mouse anti-CD52 and rabbit anti-Siglec-10 and subsequently stained with anti-mouse Alexa Fluor 488 and anti-rabbit Cy5.

## Slide 7
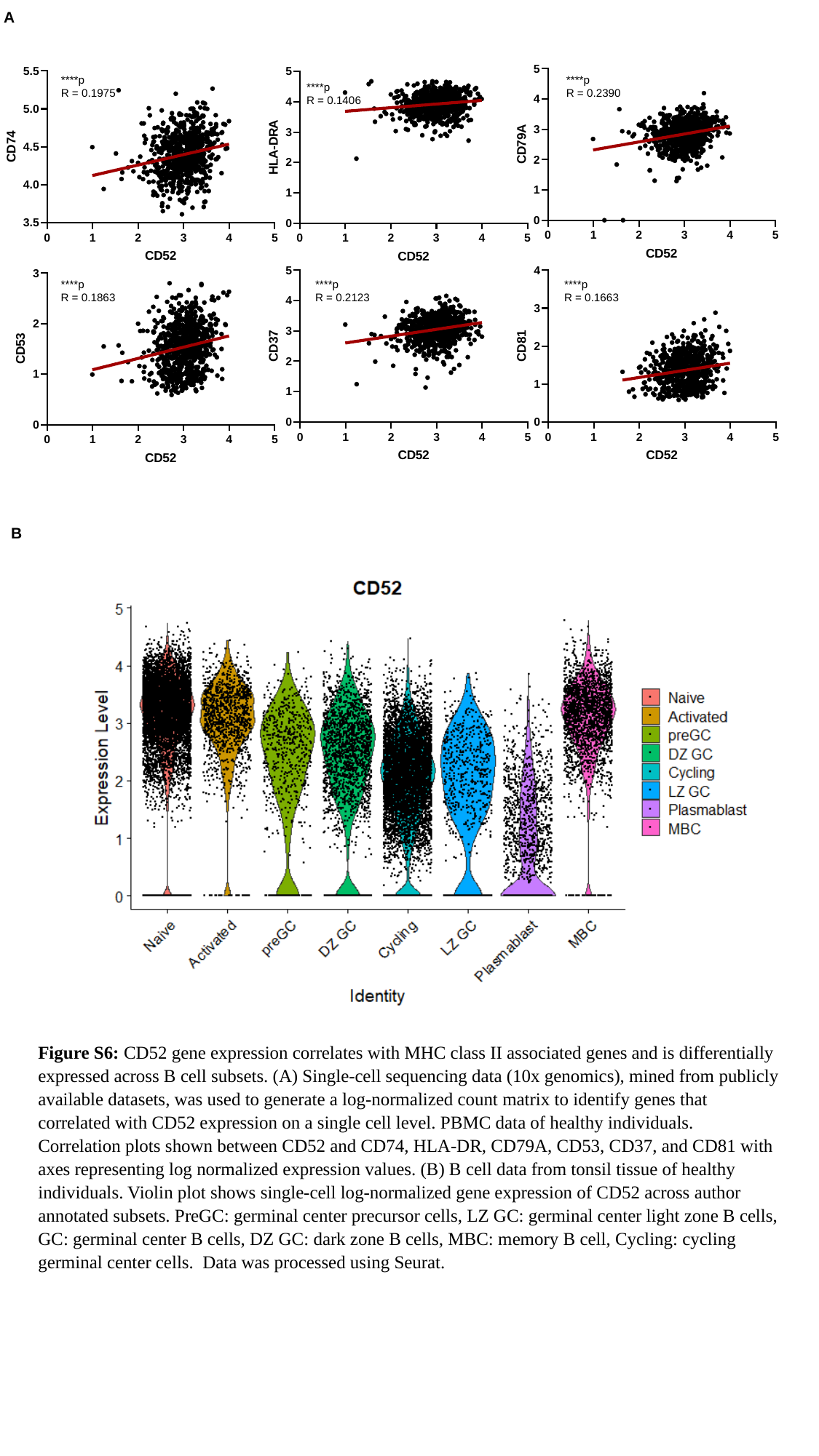

A
B
Figure S6: CD52 gene expression correlates with MHC class II associated genes and is differentially expressed across B cell subsets. (A) Single-cell sequencing data (10x genomics), mined from publicly available datasets, was used to generate a log-normalized count matrix to identify genes that correlated with CD52 expression on a single cell level. PBMC data of healthy individuals. Correlation plots shown between CD52 and CD74, HLA-DR, CD79A, CD53, CD37, and CD81 with axes representing log normalized expression values. (B) B cell data from tonsil tissue of healthy individuals. Violin plot shows single-cell log-normalized gene expression of CD52 across author annotated subsets. PreGC: germinal center precursor cells, LZ GC: germinal center light zone B cells, GC: germinal center B cells, DZ GC: dark zone B cells, MBC: memory B cell, Cycling: cycling germinal center cells. Data was processed using Seurat.

## Slide 8
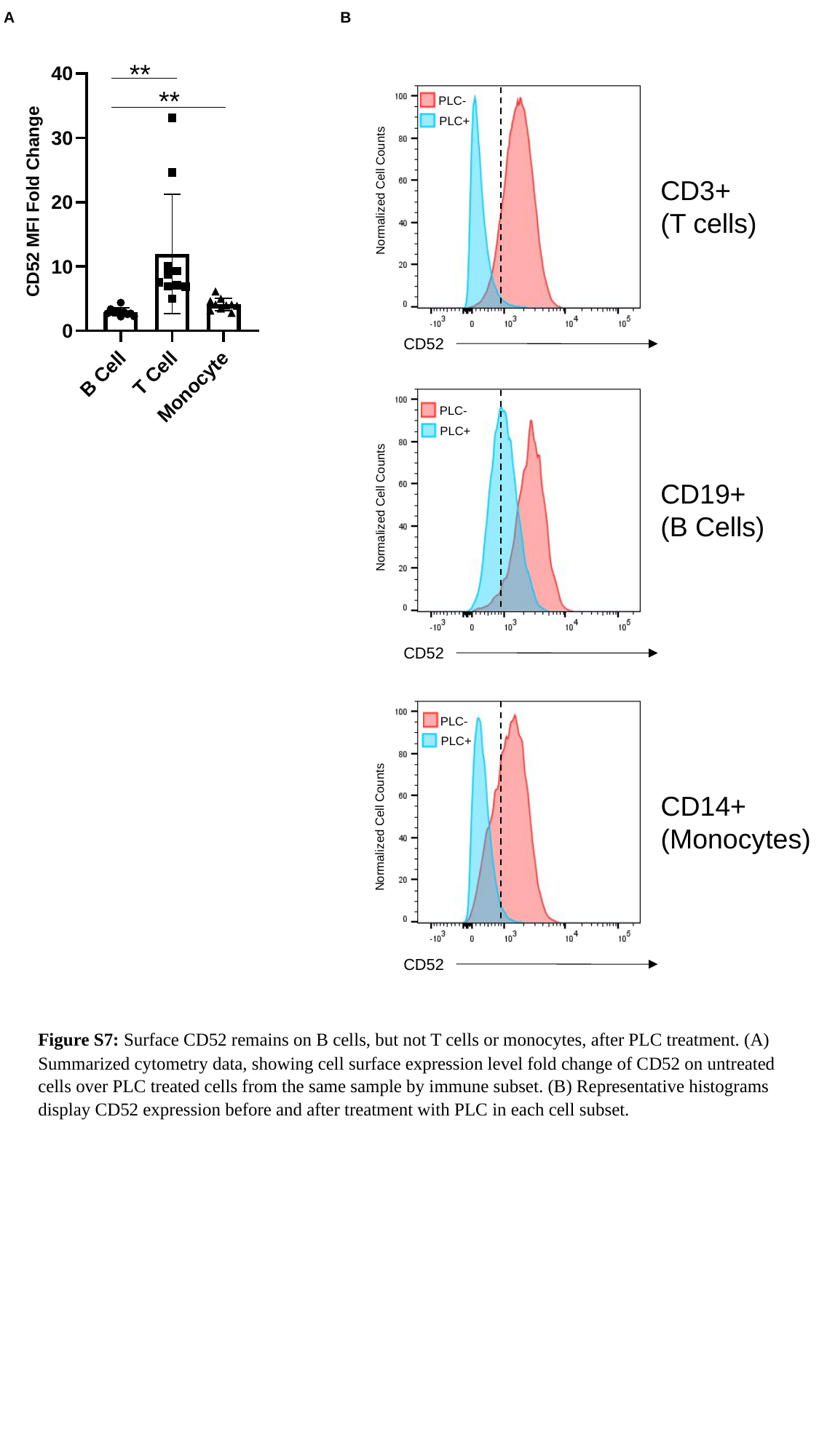

A
B
**
**
PLC-
PLC+
CD3+
(T cells)
Normalized Cell Counts
CD52
PLC-
PLC+
CD19+
(B Cells)
Normalized Cell Counts
CD52
PLC-
PLC+
CD14+
(Monocytes)
Normalized Cell Counts
CD52
Figure S7: Surface CD52 remains on B cells, but not T cells or monocytes, after PLC treatment. (A) Summarized cytometry data, showing cell surface expression level fold change of CD52 on untreated cells over PLC treated cells from the same sample by immune subset. (B) Representative histograms display CD52 expression before and after treatment with PLC in each cell subset.

## Slide 9
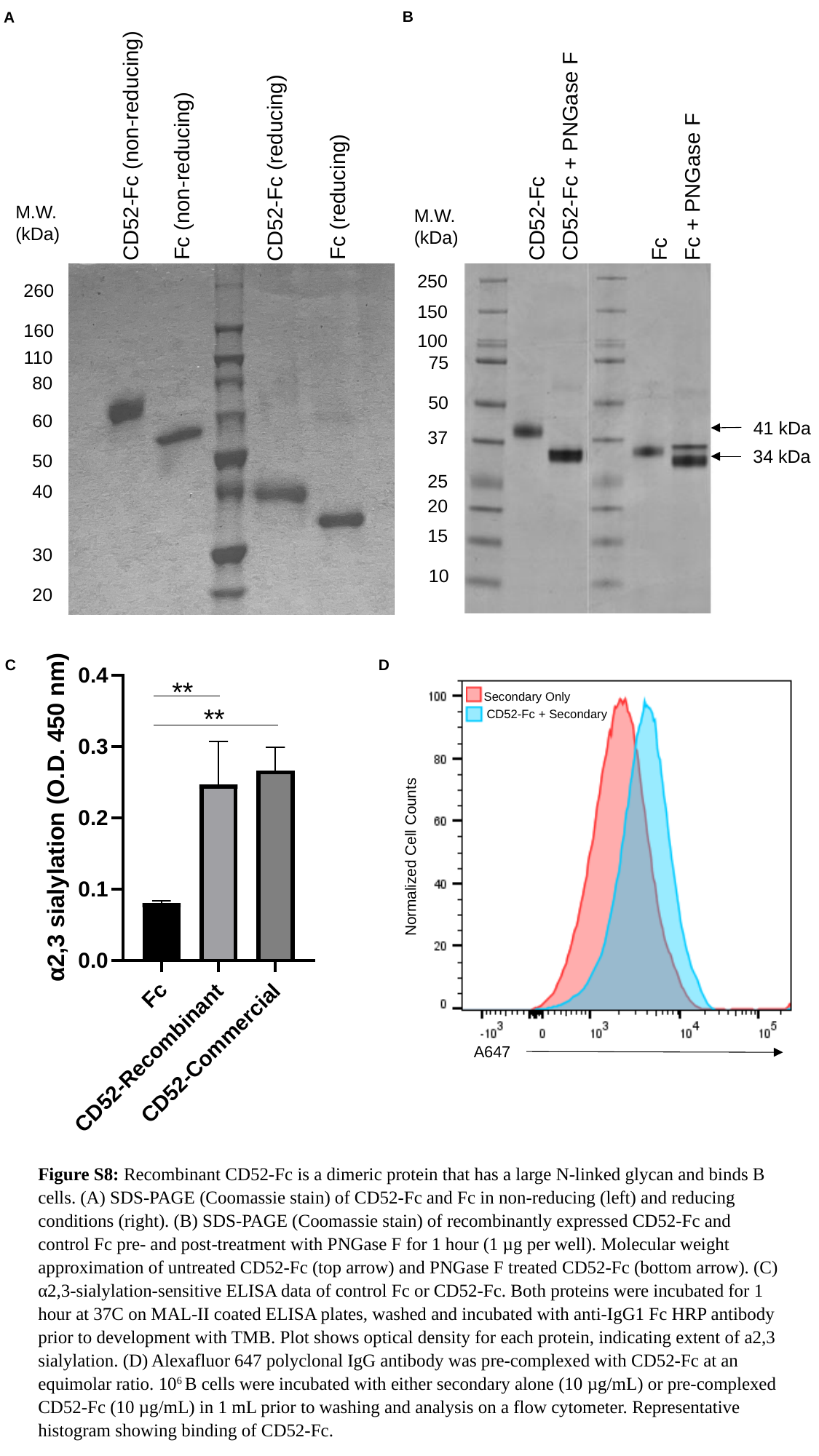

B
A
CD52-Fc (non-reducing)
CD52-Fc + PNGase F
CD52-Fc (reducing)
Fc (non-reducing)
Fc + PNGase F
Fc (reducing)
M.W.
(kDa)
M.W.
(kDa)
CD52-Fc
Fc
250
260
150
160
100
110
75
80
50
60
41 kDa
37
34 kDa
50
25
40
20
15
30
10
20
C
D
**
Secondary Only
**
CD52-Fc + Secondary
Normalized Cell Counts
A647
Figure S8: Recombinant CD52-Fc is a dimeric protein that has a large N-linked glycan and binds B cells. (A) SDS-PAGE (Coomassie stain) of CD52-Fc and Fc in non-reducing (left) and reducing conditions (right). (B) SDS-PAGE (Coomassie stain) of recombinantly expressed CD52-Fc and control Fc pre- and post-treatment with PNGase F for 1 hour (1 µg per well). Molecular weight approximation of untreated CD52-Fc (top arrow) and PNGase F treated CD52-Fc (bottom arrow). (C) α2,3-sialylation-sensitive ELISA data of control Fc or CD52-Fc. Both proteins were incubated for 1 hour at 37C on MAL-II coated ELISA plates, washed and incubated with anti-IgG1 Fc HRP antibody prior to development with TMB. Plot shows optical density for each protein, indicating extent of a2,3 sialylation. (D) Alexafluor 647 polyclonal IgG antibody was pre-complexed with CD52-Fc at an equimolar ratio. 106 B cells were incubated with either secondary alone (10 µg/mL) or pre-complexed CD52-Fc (10 µg/mL) in 1 mL prior to washing and analysis on a flow cytometer. Representative histogram showing binding of CD52-Fc.

## Slide 10
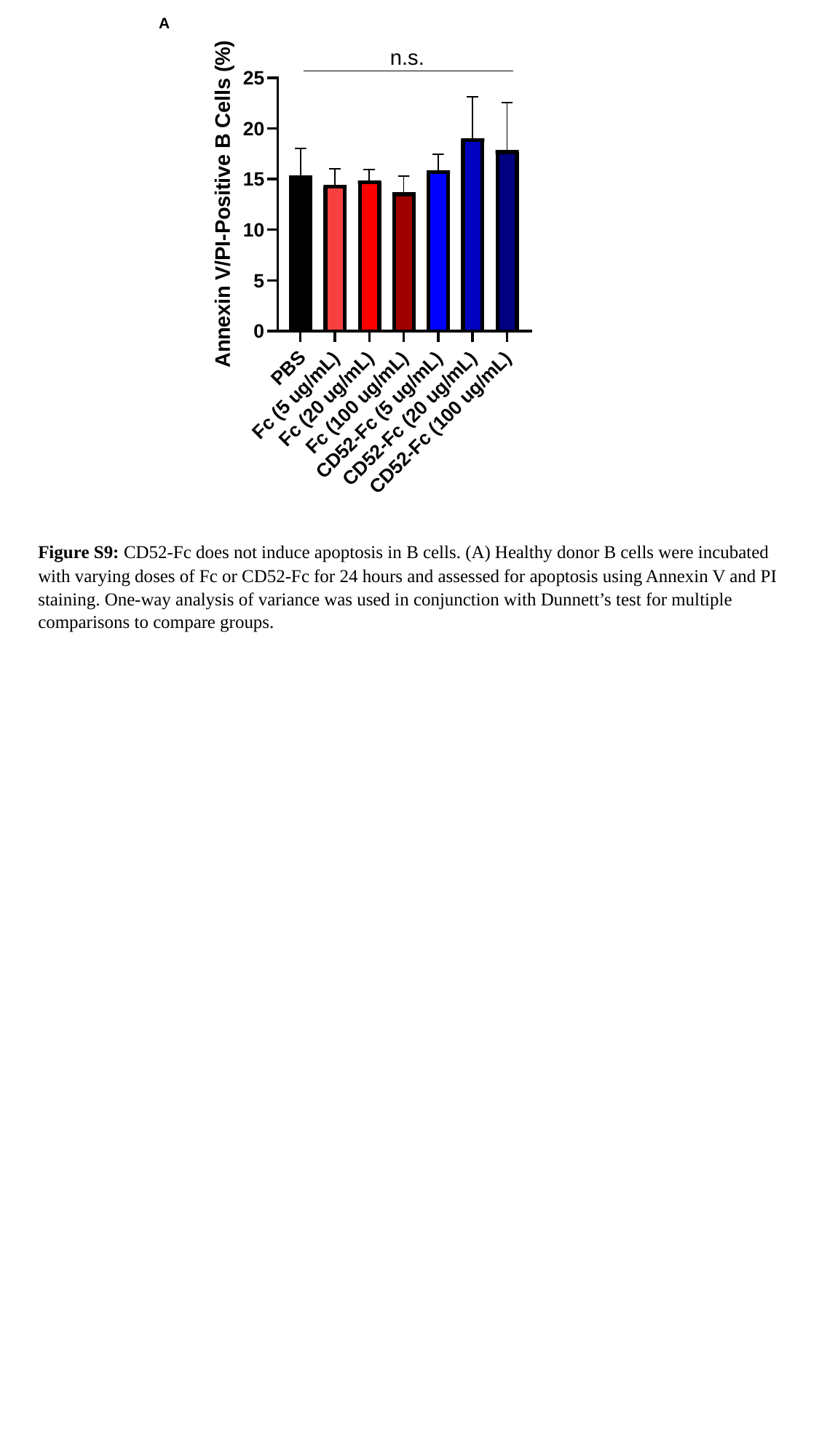

A
n.s.
Figure S9: CD52-Fc does not induce apoptosis in B cells. (A) Healthy donor B cells were incubated with varying doses of Fc or CD52-Fc for 24 hours and assessed for apoptosis using Annexin V and PI staining. One-way analysis of variance was used in conjunction with Dunnett’s test for multiple comparisons to compare groups.

## Slide 11
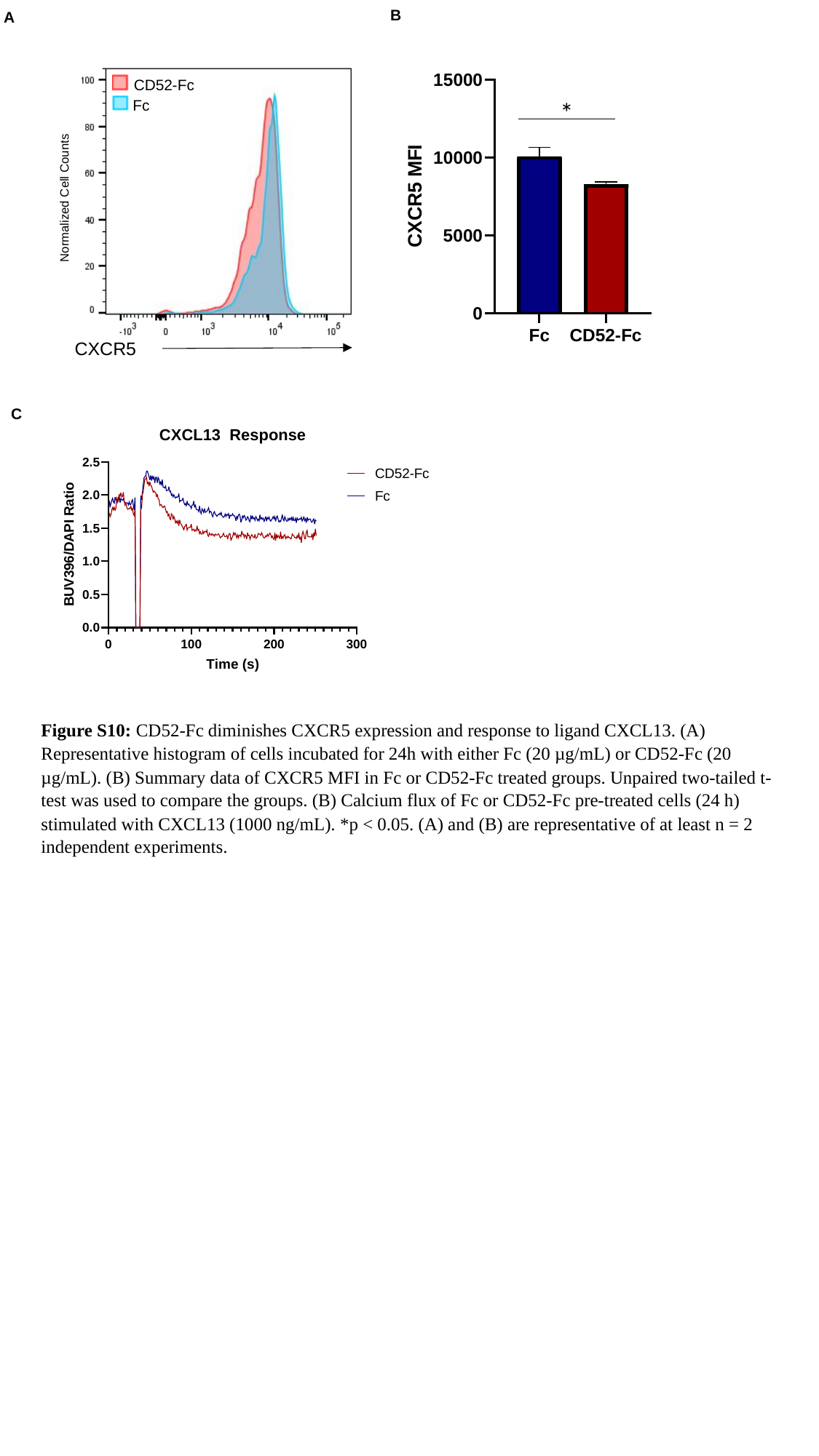

B
A
CD52-Fc
*
Fc
Normalized Cell Counts
CXCR5
C
Figure S10: CD52-Fc diminishes CXCR5 expression and response to ligand CXCL13. (A) Representative histogram of cells incubated for 24h with either Fc (20 µg/mL) or CD52-Fc (20 µg/mL). (B) Summary data of CXCR5 MFI in Fc or CD52-Fc treated groups. Unpaired two-tailed t-test was used to compare the groups. (B) Calcium flux of Fc or CD52-Fc pre-treated cells (24 h) stimulated with CXCL13 (1000 ng/mL). *p < 0.05. (A) and (B) are representative of at least n = 2 independent experiments.
